# The Impact of Polyethylene Glycol Lipid Anchors on the Physicochemical Properties, Protein Corona, Function, and Biodistribution of Lipid Nanoparticles

**DOI:** 10.1021/acsnano.5c19757

**Published:** 2026-03-18

**Authors:** Chuan-En Lu, Kai Liu, Audrey Gallud, Viktoriia Meklesh, Lisbeth Thorup Ravnkilde, Juna Santos, Filipa Dias Louro, Tasso Miliotis, Marco A. Alfonzo-Mendez, Joanna Rejman, Luca Panariello, Hanna M.G. Barriga, Molly M. Stevens, Fredrik Höök, Marianna Yanez Arteta, Johan Ulander, Suzy Jones, Alan Sabirsh

**Affiliations:** 1 Advanced Drug Delivery, Pharmaceutical Sciences, R&D, 128698 AstraZeneca, Gothenburg 43183, Sweden; 2 Data Science & Modelling, Pharmaceutical Sciences, R&D, 128698 AstraZeneca, Gothenburg 43183, Sweden; 3 Research and Early Development, Cardiovascular, Renal and Metabolism, BioPharmaceuticals R&D, 128698 AstraZeneca, Gothenburg 43183, Sweden; 4 Department of Medical Biochemistry and Biophysics, Karolinska Institutet, Stockholm SE-171 77, Sweden; 5 Division of NanoBiotechnology, Department of Protein Science, SciLifeLab, KTH Royal Institute of Technology, Stockholm 17165, Sweden; 6 Department of Physiology, Anatomy and Genetics, Department of Engineering Science, Kavli Institute for Nanoscience Discovery, 6396 University of Oxford, Oxford OX1 3QU, United Kingdom; 7 Department of Physics, Division of Nano and Biophysics, Chalmers University of Technology, Gothenburg 41296, Sweden

**Keywords:** lipid nanoparticle, protein corona, Raman spectroscopy, mass spectroscopy, polyethylene glycol, lipid chemistry

## Abstract

When introduced into biological systems, the function and biodistribution of lipid nanoparticles (LNPs) are affected by the biomolecular coronas they acquire. Corona composition is determined by the biophysical and chemical properties of the particles and the contents of the biofluids. Polyethylene glycol (PEG) polymers, anchored using lipids that partition into LNPs during formulation, are key to LNP stability in circulation. It is, however, not well-studied how different PEG-lipid anchors, with different acyl chain lengths, headgroup/linker chemistries, and desorption rates (PEG “shedding” from nanoparticles) can affect corona composition and LNP function. Here, we examined how common PEG-lipid anchors affect (1) *in vivo* biodistribution in C57BL/6 mice, (2) corona content (using mass spectrometry-based proteomics), (3) LNP biophysical characteristics (using single-particle automated Raman trapping analysis (SPARTA^Ⓡ^)), and (4) *in vitro* particle function (using cellular uptake and cargo delivery assays). Following nanoparticle formulation with clinically approved, commonly used PEG anchors, we found that the LNP biodistribution is strongly impacted, particularly in the liver, spleen, bone marrow, and lung. We then tested a wide range of lipid ratio combinations using high-throughput evaluation *in vitro.* Despite being minor LNP components (by molar ratio), the PEG-lipid anchors strongly impact the chemical characteristics, corona content, and particle function. These findings reveal structure–activity relationships between PEG-lipid anchor chemistry and functional LNP biodistribution, with implications for rational LNP design.

## Graphical abstract

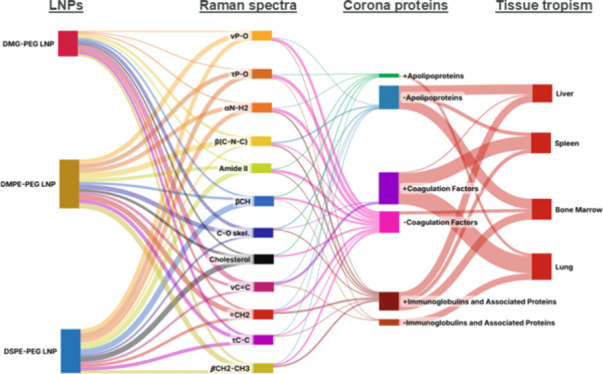

## Introduction

Lipid nanoparticles (LNPs) are widely recognized as clinically viable drug delivery systems for nucleic acid therapeutics. Enhancing the tissue-specific targeting of LNPs would further increase their utility in extrahepatic applications through systemic administration. There are two approaches commonly used to achieve this. First, LNPs can be engineered for targeted drug delivery by adding low-molecular-weight binders, full antibodies, or antibody fragments to particle surfaces.^1^ Second, as an endogenous targeting tactic, the composition of the LNPs can be adjusted to modulate the bimolecular coronation process that occurs when LNPs interact with biological fluids like blood.^2,3^ This corona, a dynamic and complex structure containing proteins, lipids, and carbohydrates, plays a crucial role in influencing where the particles go and how long they stay in circulation.^4–6^ Corona also impacts cellular uptake, endosomal escape, and cargo delivery of LNPs *in vitro* and *in vivo*.^7^

Previous changes in LNP lipid composition mainly focused on ionizable cationic lipids or additional permanently charged lipids.^8,9^ Polyethylene glycols have received less attention, regarding their specific roles in LNP distribution and mechanisms of action. For use in LNPs and other lipid-based nanoparticles, PEGs are attached covalently to lipid anchors to form PEGylated lipids. PEG-lipids, introduced to condense and stabilize these hydrophobic particles, are incorporated during microfluidic LNP formulation and usually represent less than 1.5% molar ratio of the LNP composition.^10^ Following the maturation of LNPs, the water-facing PEG-lipids are positioned close to the surface of the LNPs, making the anchor moieties potentially relevant for defining LNP surface characteristics.^11^ When exposed to biofluids, the PEG-lipids gradually dissociate (also known as PEG shedding) from the LNP surfaces. The rate of disassociation depends on lipid anchor properties that can be modulated by changing the length, saturation, and branching of the lipid anchor acyl chain.^12^ For example, the longer the acyl chain, the stronger the PEG-lipids are anchored on the LNP surface.^13^ PEG-lipids are believed to extend particle circulation times by altering corona formation and corona-related LNP clearance.^14^

Previous studies indicate that LNPs with different PEG-lipid anchors perform differently *in vitro* and *in vivo.* ^13,15^ However, the relationships between LNP composition, the chemical characteristics of the resulting particles, corona formation, and particle function are not well-explored. In this study, groups of compositionally diverse LNPs, each containing one of three commonly used PEG-lipids, 1,2-dimyristoyl-*sn*-glycerol-PEG2000 (DMG-PEG), 1,2-dimyristoyl-*sn*-glycero-3-phosphoethanolamine-PEG2000 (DMPE-PEG), and 1,2-distearoyl-*sn*-glycero-3-phosphoethanolamine (DSPE-PEG), were evaluated in detail (Figure 1A). DMG-PEG contains two myristoyl (C14) acyl chains and a glycerol backbone with ester linkages; DMPE-PEG has identical myristoyl acyl chains but an ethanolamine headgroup with a phosphate linker. DSPE-PEG features two stearoyl (C18) acyl chains with the same headgroup and phosphate linker, and owing to the longer acyl chain length, DSPE-PEG is expected to be more strongly associated with the LNP.

**Figure 1:**
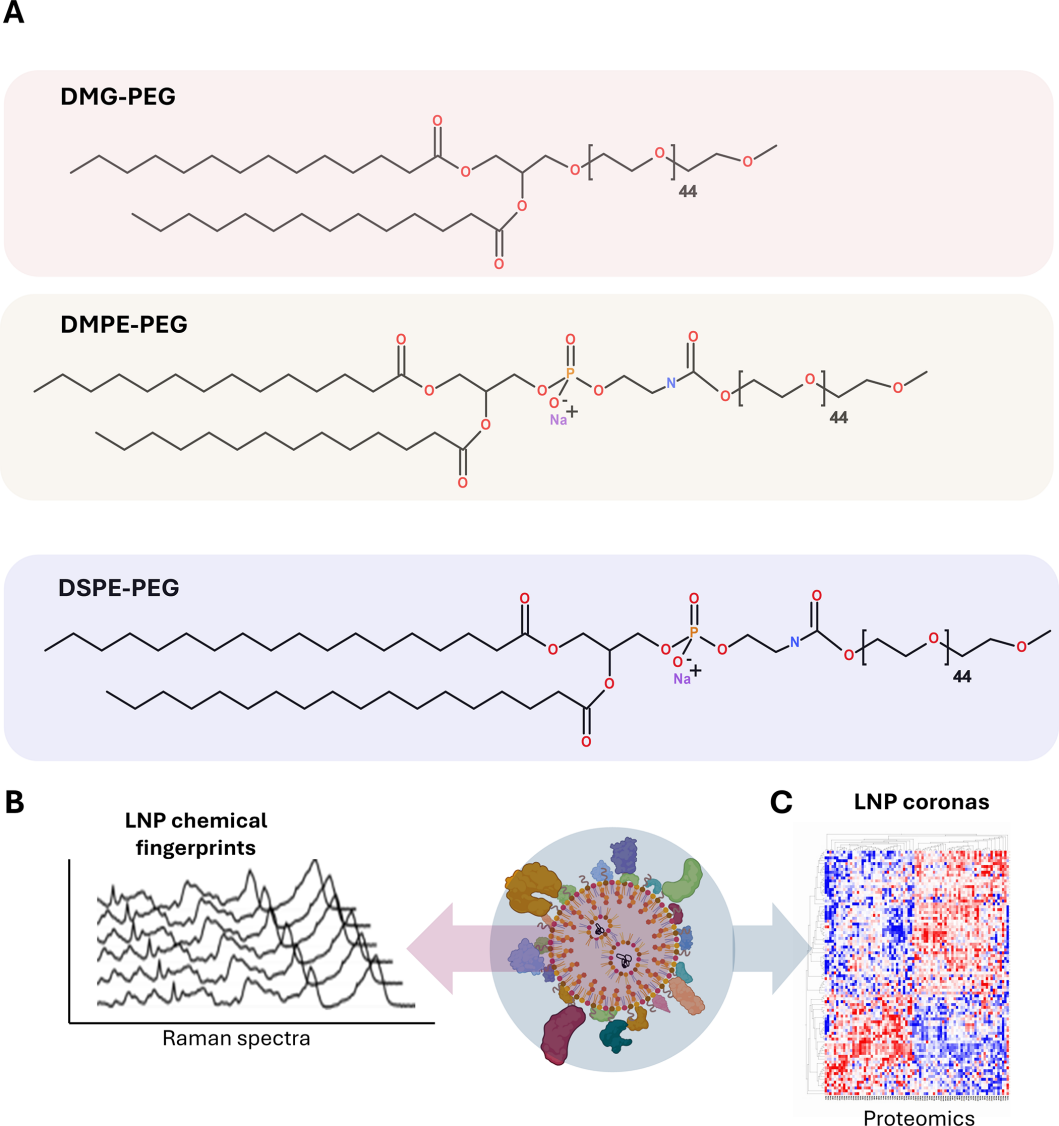
Comprehensive physicochemical and coronal characterization of LNPs with diverse PEG-lipids. (A) Chemical structures of PEG-lipid conjugates used in this study: DMG-PEG, DMPE-PEG, and DSPE-PEG. (B) Single-particle chemical fingerprints of intact LNPs (without corona proteins) acquired by SPARTA^Ⓡ^, highlighting lipid-anchored-dependent spectral features. (C) Protein corona composition formed in mouse serum, quantified by mass spectrometry.

This work links spectroscopic chemical signatures from intact LNPs with different PEG-lipid anchors to corona formation and both *in vitro* and *in vivo* function. We used both single-particle and ensemble measurement methods to evaluate average size, size distribution, and cargo encapsulation of each formulation. Single-particle automated Raman trapping analysis (SPARTA^Ⓡ^) was also employed to measure chemical fingerprints of individual LNPs (Figure 1B). The same set of formulations was also added to biofluids and then retrieved to determine, using proteomic mass spectroscopy, the composition of the particle coronas. *In vivo* functional biodistribution was also evaluated (Figure 1C). Finally, a wider range of formulations, 24 LNPs for each PEG-lipid anchor, were evaluated functionally *in vitro*; these LNPs differed in the relative ratios of additional lipids, while the encapsulated Cy5-labled-eGFP mRNA cargos remained constant across all samples. The PEG-lipid anchors were found to have large effects on Raman spectra, corona content, and *in vitro and in vivo* function. Remarkably, the chemical spectroscopic information correlated with both the corona content and particle function, suggesting that predictive models of LNP interactions with biomolecules and LNP distribution may be possible using only physical measurements of nanoparticle formulations. By linking spectroscopic fingerprints with coronal proteomics and *in vitro*/*in vivo* outcomes, we show that subtle anchor-dependent differences encode predictable biodistribution and efficacy patterns, with DMPE-PEG favoring liver and bone marrow activity and DSPE-PEG attenuating early function. These results show that PEG-lipid anchors are not passive excipients but active determinants of LNP surface chemistry, corona composition, and tissue-specific function, highlighting that physical and molecular signatures can serve as predictive markers for rationally designing LNPs with tunable tropism by using minimal empirical screening.

## Results and Discussion

### LNP Dosing and Functional Evaluation: The Impact of PEG-Lipid Anchors

We first asked how the chemical nature of PEG-lipid anchors influences the physicochemical properties and early functional delivery of mRNA-loaded lipid nanoparticles. To isolate the effect of the PEG-lipid, we formulated LNPs using the same base composition as Onpattro (Dlin-MC3-DMA/DPSC/cholesterol/PEG-lipid, 50:10:38.5:1.5 mol %),^16^ varying only the PEG-lipid anchor: DMG-PEG, DMPE-PEG, or DSPE-PEG. All three formulations encapsulated firefly luciferase (FLuc) mRNA with comparable mRNA encapsulation efficiency (92.9–93.8%) and polydispersity index (PDI) (0.19–0.25). Particle size was measured by two orthogonal methods—dynamic light scattering (DLS; Table s1) and small-angle X-ray scattering (SAXS; Table s2). DMPE-PEG or DSPE-PEG of LNPs fell within a similar size range (approximately 100 nm), with DMG-PEG LNPs roughly 20% larger.

PEG-lipid anchors differ in acyl chain length and desorption kinetics (PEGs with DMG and DMPE anchors are more readily desorbed (shed) from particle surfaces, whereas DSPE increases PEG retention), so the current understanding is that PEGs with higher desorption rates (“sheddable” PEGs) enhance hepatic uptake while nonshedding PEGs can reduce early liver function. To test this in a controlled *in vitro* setting, we assessed transfection in Hepa1-6 mouse hepatoma cells with LNPs preincubated in mouse serum to promote protein corona formation. At 24 h, cells dosed with DMG-PEG and DMPE-PEG LNPs showed approximately an order-of-magnitude higher luciferase signal than cells dosed with DSPE-PEG LNPs, with DMPE-PEG LNPs yielding the highest activity (Figure 2A).

**Figure 2:**
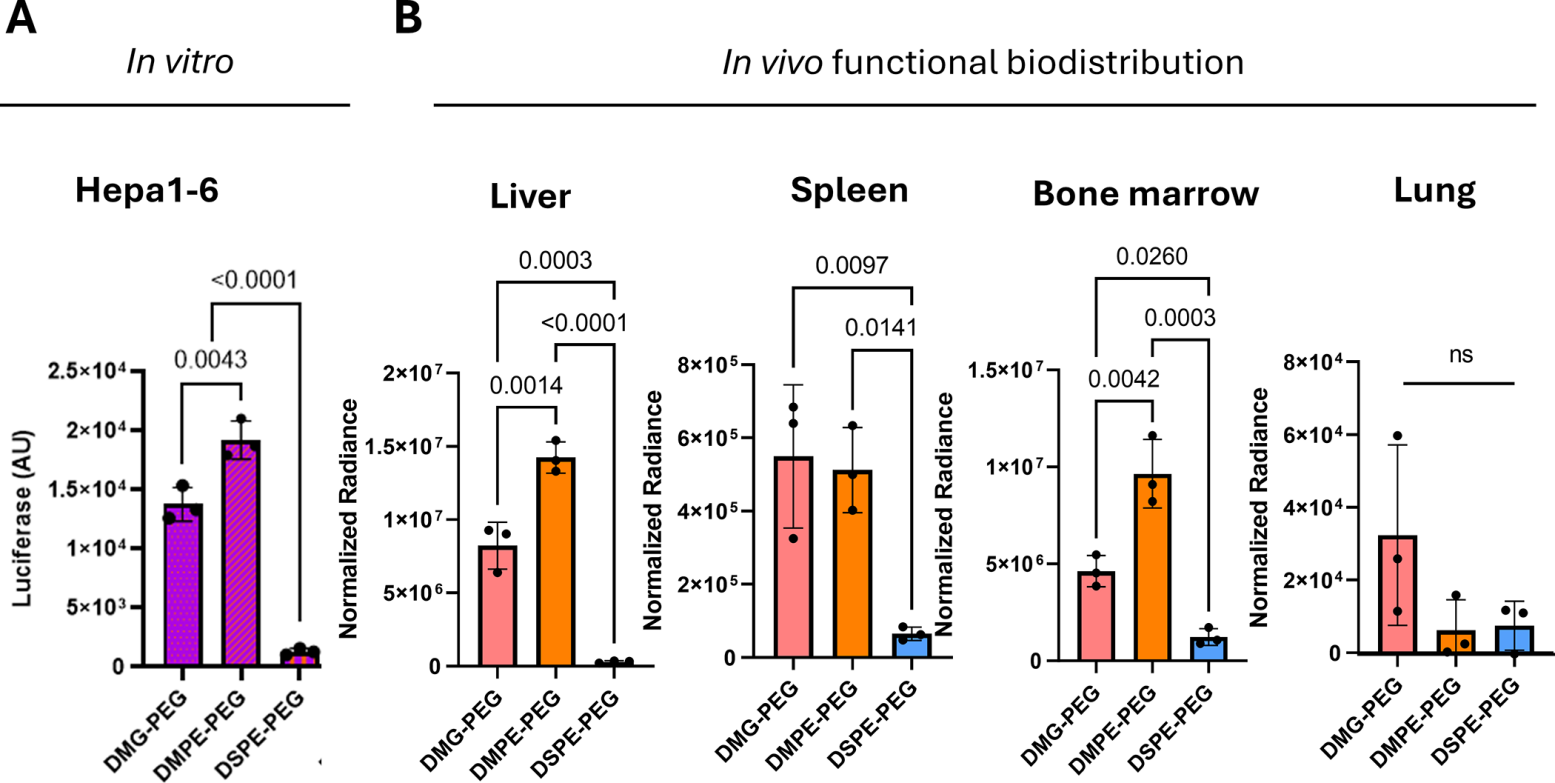
Function and distribution of LNPs formulated with different PEG-lipid anchors. (A) *In vitro* luciferase assay on the Hepa1-6 cell line with mouse serum. LNP cargo: FLuc-encoded mRNA. *p* values were listed in the figure. (B) *In vivo* functional biodistribution study illustrating different tissue tropism of LNPs with different lipid-PEG. Color-coded by PEG-lipid anchor: DMG-PEG (red), DMPE-PEG (orange), and DSPE-PEG (blue). (*n* = 3, one-way ANOVA, numbers represent *p* values, ns = not significant).

We next evaluated early *in vivo* expression following systemic LNP administration, sacrificing mice at 6 h to capture initial delivery. The functional hierarchy observed *in vitro* was recapitulated *in vivo*: DMPE-PEG LNPs produced the strongest luciferase expression in the liver (Figure 2B), consistent with preferential hepatic uptake under conditions favoring PEG shedding. DMG-PEG LNPs showed intermediate activity, whereas the larger, more surface-retentive DSPE-PEG LNPs were largely inactive at this early time point. Outside the liver, we detected a similar pattern in the bone marrow, with DMPE-PEG > DMG-PEG ≫ DSPE-PEG. The spleen was less discriminating: DMG-PEG and DMPE-PEG LNPs performed comparably, although absolute signals were ∼100-fold lower than in the liver and the bone marrow. Lung expression was minimal for all formulations at 6 h.

### LNPs with Different PEG-Lipid Anchors Have Unique Protein Corona Profiles

Next, we wanted to address what drives the differential organ tropism in mice injected with distinct PEG-lipid LNPs. We hypothesized that once they enter the bloodstream, these LNPs are decorated with coronas with unique protein compositions. To test this, we used a proteomic approach. Briefly, we incubated the LNPs with mouse serum to mimic the corona formation in circulation and then enriched the LNP population, using magnetic beads conjugated with anti-PEG antibodies, prior to obtaining proteomic corona profiles.^9^ The three different PEG-lipid anchors yielded robust and distinct quantitative proteomic profiles, with many proteins exhibiting statistically significant differences in terms of quantity in label-free quantification (LFQ) (Figure 3A). For a global view, the top 20 proteins (Figure 3B) were defined by averaging intensity-based absolute quantification (iBAQ) intensities for each protein across all samples from all three anchor groups and ranking the resulting global means. DMG-PEG and DSPE-PEG showed broadly similar top 20 patterns, whereas DMPE-PEG diverged. Total protein loads were modestly higher for DMG-PEG than for DMPE-PEG or DSPE-PEG, without reaching statistical significance (Figure 3C), which may reflect the slightly larger size (and therefore the available surface area) of DMG-PEG LNPs observed by DLS and SAXS.

**Figure 3:**
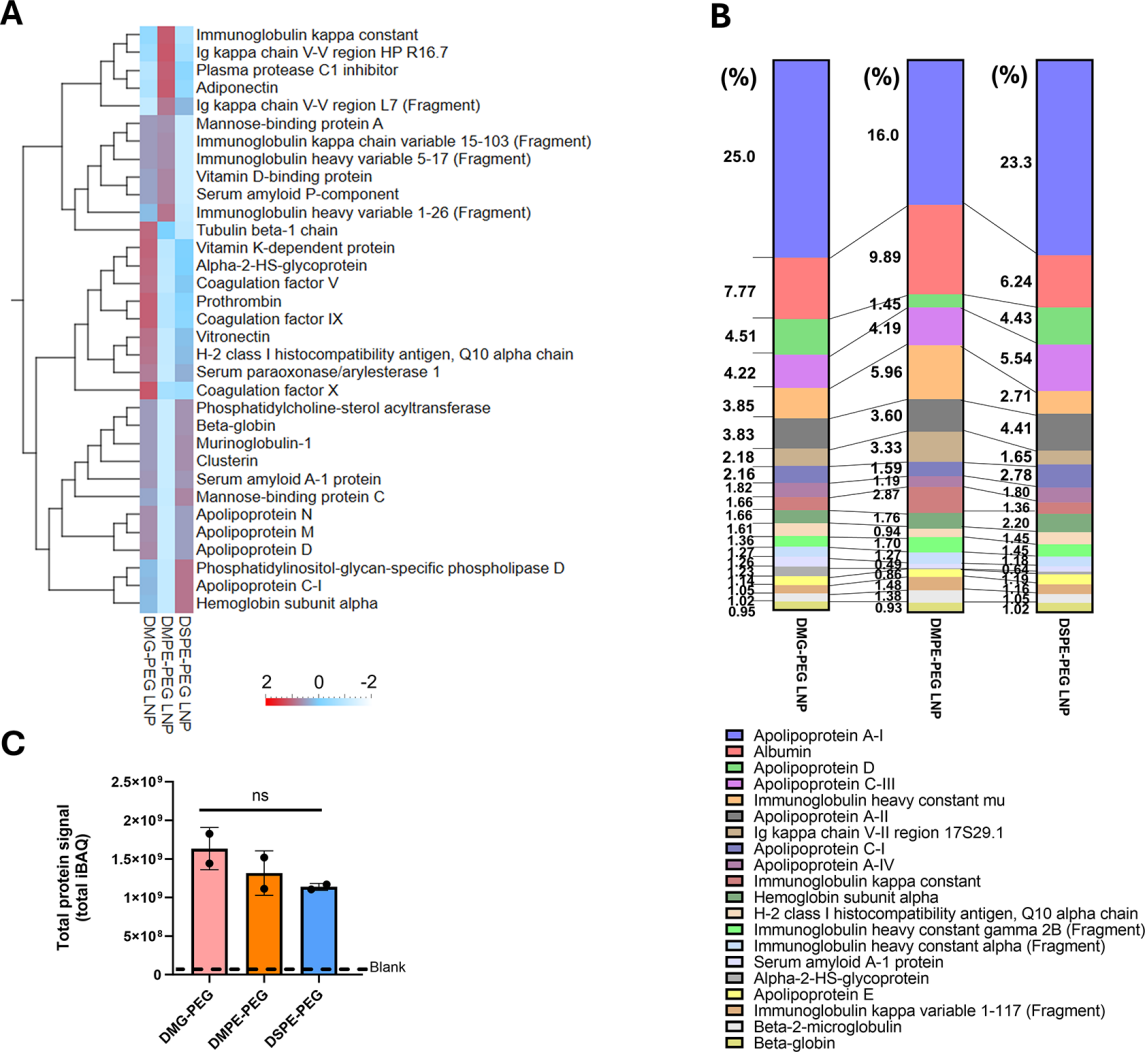
*Ex vivo* LNP corona profiling across LNPs with different PEG-lipids. (A) Proteomic heatmap of identified LNP corona proteins with statistical differences between LNPs with different PEG-lipid anchors (values based on log_2_ LFQ, average of duplicated data, *p* < 0.05, one-way ANOVA). (B) Top 20 corona proteins computed across all three anchor groups: for each protein, iBAQ intensities were averaged over all samples (duplicates) from all groups and then ranked globally; values show the percentage contribution of each protein. (C) Total iBAQ signals of isolated LNP corona fractions and “blank” captures performed on 1% mouse serum without LNPs using an immunomagnetic separation workflow.

Although the limited number of LNP types evaluated here precludes predictive modeling, the two anchors associated with liver, bone, and spleen activities (DMG-PEG and DMPE-PEG) share category-level corona features that may contribute to function. Among protein categories with statistically significant differences, these three predominant categories emerged—apolipoproteins (Figure 4A), coagulation factors (Figure 4B), and immunoglobulins (Figure 4C)—with anchor-specific fingerprints within each category. Apolipoproteins were comparatively more abundant on DSPE-PEG and DMG-PEG LNPs, whereas DMPE-PEG LNPs showed lower levels of several apolipoproteins.

**Figure 4:**
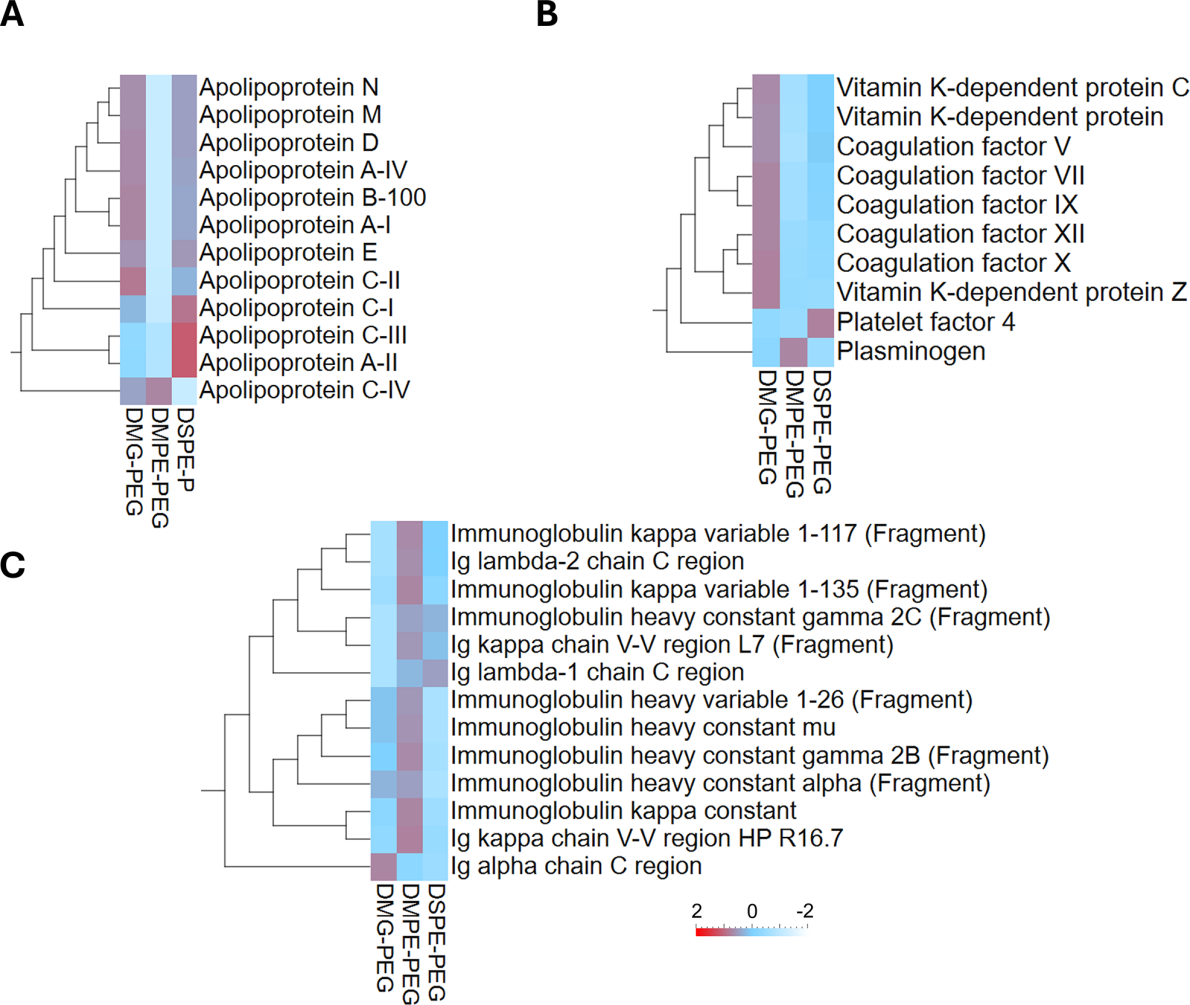
Representative members of major corona protein clusters. Panels A–C show detailed views, based on log_2_ LFQ, average of duplicate corona samples, of three protein categories, (A) apolipoproteins, (B) coagulation factors, and (C) immunoglobulins.

Apolipoproteins ranked among the top 20 proteins for all formulations, consistent with prior reports that LNPs acquire lipoprotein-associated proteins in serum.^17–20^ However, while apolipoprotein E (ApoE) is often proposed to adsorb strongly and mediate hepatic delivery, our data show a relative enrichment of ApoE on DSPE-PEG LNPs and for DMG-PEG relative to DMPE-PEG LNPs, even though DMPE-PEG LNPs showed better hepatic function *in vivo*. The explanation could be that apolipoproteins are prevalent across all of the LNPs evaluated, and the anchor-mediated differences are modest in the context of high baseline adsorption based on iBAQ values (Figure 3B). This high background of apolipoprotein binding across three groups likely explains why comparing small quantitative shifts (e.g., ApoE and ApoC-III) can be challenging and why functional differences are better captured by multivariate patterns across protein categories. This also suggests that alternative mechanisms (e.g., immunoglobulin-mediated interactions and anchor-dependent surface chemistry affecting receptor engagement or clearance kinetics) may contribute to DMPE-PEG performance in the liver and bone marrow.

Furthermore, DMG-PEG LNPs were enriched with vitamin K-dependent clotting factors including prothrombin (F2), vitamin K-dependent proteins, and coagulation factors V, VII, IX, X, and XII. DMG-PEG LNPs also recruited vitronectin (VTN), which is notable for its reported involvement in lung targeting.^20,21^ While not significantly better in our study, the DMG-PEG LNPs that recruited VTN were the best performing LNPs in the lungs. Finally, DMPE-PEG LNPs preferentially recruited immunoglobulin proteins to their surfaces but not coagulation factors or apolipoproteins.

Conversely, proteins that are absent from the coronas around these particles could also be of interest. The particles that were the most functionally active in the liver (DMPE-PEG LNPs) had the lowest levels of various apolipoproteins, while the less active DMG-PEG LNPs recruited more apolipoproteins and yet displayed reduced liver activity. One plausible explanation is anchor-dependent surface organization—our molecular dynamics (MD) simulations showed that DMG-PEG can promote PEG clustering (Figure s1), sterically hindering productive engagement of cell-surface receptors by coronal components. Coagulation factors, on the other hand, do not seem to affect the function of LNPs in the liver, with the possible exception of platelet factor 4, which was not present in the coronas of both liver-active LNPs.

### LNPs with Different PEG-Lipid Anchors Have Different Raman Spectral Signatures

Distinct corona protein patterns like these are considered critical determinants of tissue and cell tropism,^3^ but we wanted to further explore the relationship between particle function, corona content, and the physicochemical properties of the particles, so we turned to Raman spectroscopy. Detailed spectral fingerprints of single particles from the LNP formulations, with different PEG-lipids, were obtained using single-particle automated Raman trapping analysis or SPARTA^Ⓡ^. SPARTA^Ⓡ^ is a label-free and nondestructive technique that combines optical trapping with Raman spectroscopy, typically within the wavenumber range of 400–1800 cm^–1^ to evaluate the chemical characteristics of individual particles. This approach and its automation workflow have previously been described in detail.^22,23^ Here, we apply it to PEG-lipid-dependent LNP characterization. Distinct patterns corresponding to each PEG-lipid anchor type were present, and they are visualized here using principal component analysis (PCA) (Figure 5A). These differences can be exemplified by the Raman wavenumber band between 501 and 576 cm^–1^ (① in Figure 5B) and are reflected in the PC loading plot (Figure 5C). This signal might come from the structural differences in PEG-lipid anchors,^24^ where DMPE-PEG and DSPE-PEG feature phosphate linkers while DMG-PEG contains ester linkages, demonstrating that Raman spectroscopy potentially can detect known chemical features. Additional spectral assignments to chemical entities are shown in Table s3.

**Figure 5:**
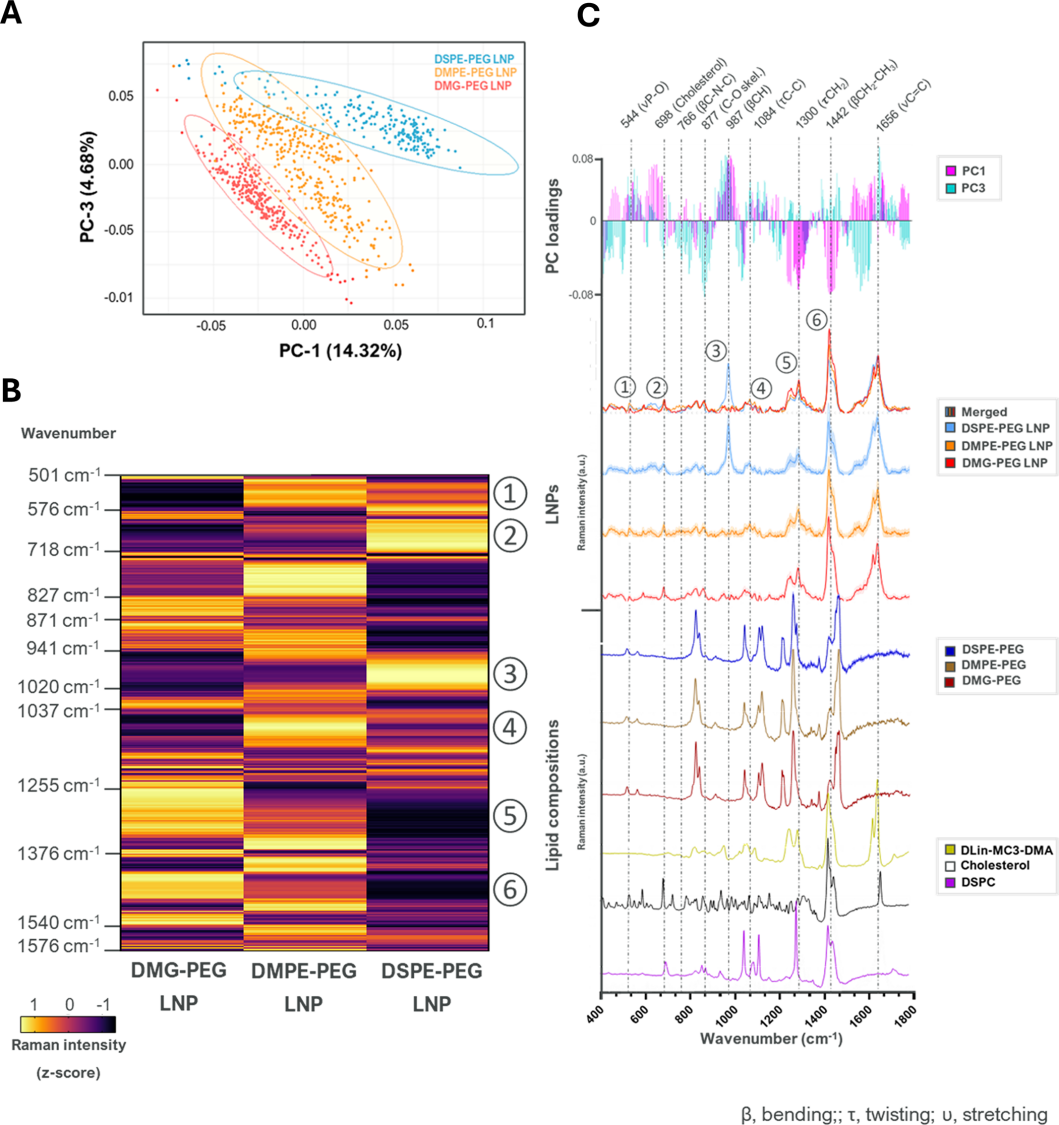
Comprehensive Raman characterization of LNPs with different PEG-lipid anchors using SPARTA^Ⓡ^. SPARTA^Ⓡ^ combines optical trapping and confocal Raman spectroscopy with automated trap recognition to acquire label-free chemical fingerprints from multiple individual nanoparticles in a particle sample. The instrumentation and automation workflow are described by Penders et al.^22^ (A) PCA scatter plots of Raman spectra from three different types of LNPs. Each data point represents the spectrum from a single nanoparticle. Total numbers of acquired spectra from individual LNPs are *n* = 310, 373, and 198 for DSPE-PEG LNPs, DMPE-PEG LNPs, and DMG-PEG LNPs, respectively. (B) *Z*-score normalized LNP spectra data averaged over all particles with each type of PEG-lipid anchor. (C) PC loading plot showing components 1 and 3 followed by the corresponding Raman spectra of LNPs (mean ± SD) and their individual component lipids as references. Numbers in circles refer to spectral bands that are discussed in the main text.

Subtle conformational changes and differences in molecular distribution can be observed by using Raman spectroscopy if the relevant chemical bonds have enough space to vibrate. For example, both DMG-PEG and DMPE-PEG LNPs showed higher intensities than DSPE-PEG between the 877 to 941 cm^–1^ band assigned to the C–O group in the PEG-skeleton structure.^25^ This is notable because all three molecules have identical PEG components, which should yield similar Raman signals. Prior research suggested that lipid anchors can change the lipid packing of LNPs, shifting the PEG Raman spectra.^26^ To explore this, we performed MD simulations to see how different lipid anchors might affect the LNP surfaces. The simulations revealed PEG clusters in DMG-PEG and DMPE-PEG LNPs, while DSPE-PEG was more evenly dispersed (Figure s1A). Clustered chemical domains can boost Raman intensity,^27,28^ matching our observations for DMG and DMPE lipid anchors. The surface clustering was quantified using measurements of radial distribution, and the order was found to be DMG > DMPE > DSPE with DMG showing the largest clusters, aligning with the trend in the Raman spectroscopy data (Figure s1B). Therefore, we propose that the Raman spectral differences stem from PEG-lipid anchor effects on surface organization. Conversely, for the 576–718 cm^–1^ band ② in Figure 5B assigned to LNP cholesterol, DSPE-PEG LNPs showed higher intensity. Though the precise positions of lipids and cargos in LNPs remain uncertain, this might suggest that cholesterol is more aggregated at the surface in DSPE-PEG LNPs. This finding could be explained with SAXS data (Figure s2). SAXS analysis revealed similar structures for all LNPs, with a peak at ∼0.11 Å^–1^, but DSPE-PEG LNPs had an extra shoulder at higher *q*-values, indicating additional features such as cholesterol clusters, consistent with the Raman results.

Importantly, despite all formulations sharing similar lipids, only with different PEG anchors, Raman spectra showed distinct changes in bands ③ (1037–1255 cm^–1^), ④ (1255–1376 cm^–1^), and ⑤ (1376–1442 cm^–1^), corresponding to C–C twisting, CH_2_ twisting, and CH_2_–CH_3_ bending, respectively. These modes represent lipid tail conformations present in all LNPs, yet their anchor-specific shifts implicate PEG-anchor-driven changes in the lipid phase organization. Using the same reasoning as with the proteomic data, to look for fingerprints indicating functionally effective particles, Raman bands ④ and ⑤ are good candidates that correlate with LNP *in vitro* and *in vivo* function. The water-soluble PEG molecules also hold the lipid anchors close to the LNP surfaces, allowing these lipids to have a surprisingly large effect on the surface chemistry of the LNPs. These findings underscore the value of analyzing molecular traits in intact nanoparticles, with SPARTA^Ⓡ^ yielding useful insights from label-free particles.

### The Influence of the PEG Content on Biophysical and Chemical Properties, Corona Composition, and Cell Functionalities

To further explore and validate the observed effects, we formulated a range of LNPs with increasing levels of PEG-lipid anchors from 0.45 to 6% for each type of anchor, and these particles were evaluated functionally *in vitro* and with corona proteomics. 24 LNPs were formulated for each PEG-lipid anchor using mRNA cargo coding for GFP, with 1/10 of this cargo consisting of GFP-mRNA molecules labeled with Cy5 fluorophores. The fluorescently labeled mRNA functions as a surrogate marker for the particles and was used to quantify particle uptake by cells. As expected, across all anchors, increasing the PEG percentage correlated with reduced particle size (Figure 6A). Conversely, lowering PEG reduced steric stabilization (particle condensation) and increased aggregation, yielding larger hydrodynamic diameters and higher PDI values (Figure s5). Formulation compositions with PEG percentages below 1.5% generated large particles (above 400 nm), especially for DMG-PEG LNPs. Notably, the size–PEG relationship between 1.5% and 6% exhibited anchor-specific slopes (*R* ^2^ = 0.637 for DMG-PEG, 0.825 for DMPE-PEG, and 0.899 for DSPE-PEG), indicating that even when PEG quantities are similar the lipid anchors impose distinct constraints on particle size. In this range, DMPE-PEG formulations produced the smallest particles, DMG-PEG were intermediate, and DSPE-PEG were the largest. These trends align with SAXS evidence for anchor-dependent structural organization (Figure s2), supporting the view that differences in anchor chemistry and surface retention modulate LNP packing and effective steric stabilization.

**Figure 6:**
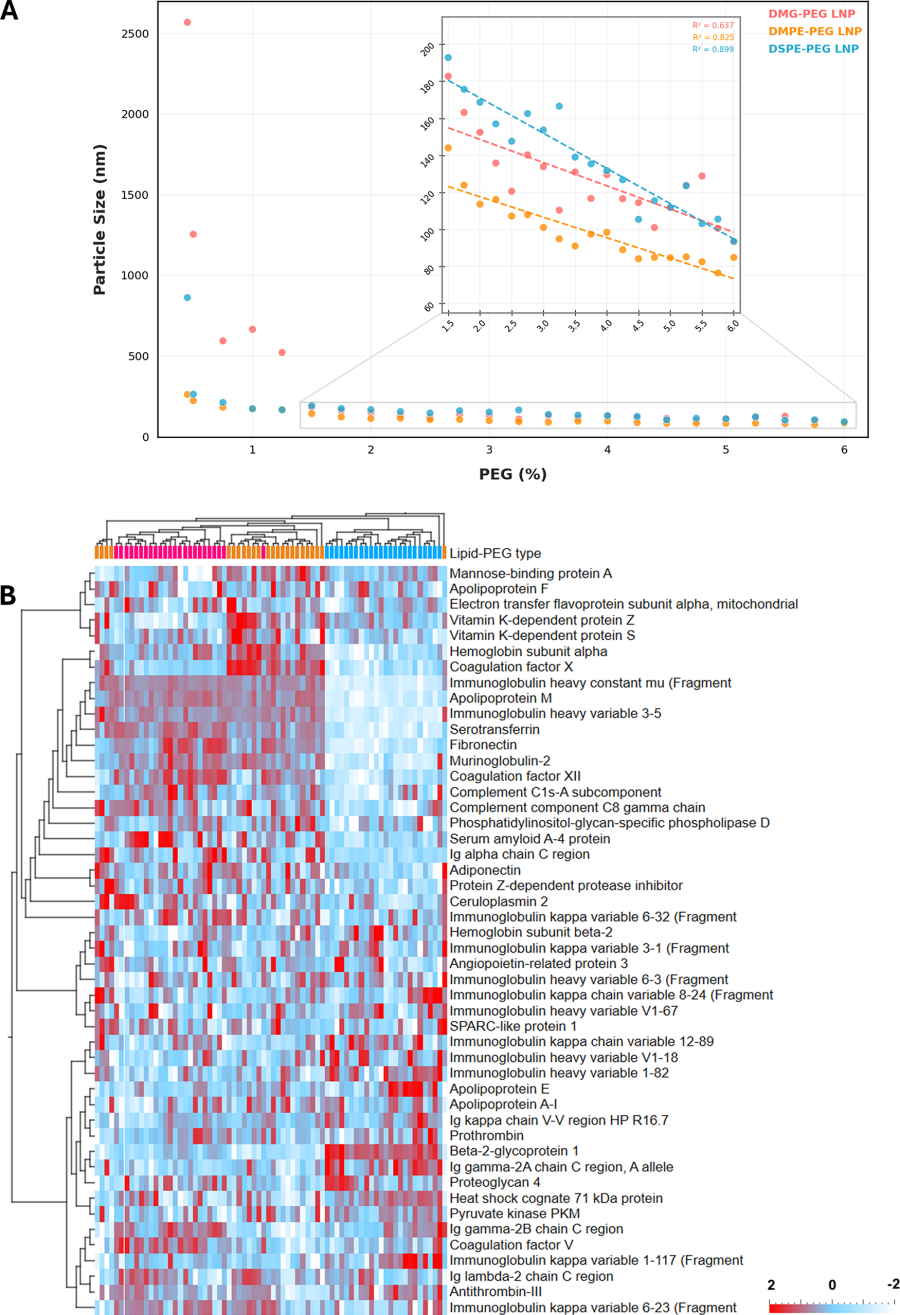
High-throughput assessment of PEG-lipid content effects on particle size and corona composition. (A) Particle size versus PEG-lipid mol % (0.45–6) across anchors (DMG-PEG, DMPE-PEG, and DSPE-PEG); 1.5–6% showed linear reductions in size with increasing PEG and anchor-specific slopes (*R* ^2^: 0.637, 0.825, and 0.899). (B) 24 distinct LNP formulations were generated using three of the PEG-lipid anchors (DMG-PEG, DMPE-PEG, and DSPE-PEG) at varying lipid-PEG molar percentages, with data representing averaged duplicates. Identified corona proteins were clustered into representative groups to reduce data set dimensionality, with each cluster represented by a single protein. Heatmap visualization displays protein corona profiles across all LNP formulations, color-coded by a PEG-lipid anchor on the top: DMG-PEG (red), DMPE-PEG (orange), and DSPE-PEG (blue). Heatmap intensity represents *Z*-scores, with red indicating higher corona content (LFQ) and blue to white indicating lower content for each protein.

In addition to the correlation between chemical and quantitative properties of PEG-lipid anchors with cell functionalities, how the quantity of PEG-lipids affects protein corona profiles was also evaluated. More than 500 proteins can be identified, and entire LNP corona profiles were normalized by the total amount of proteins per LNP. Subsequently, these proteins were clustered into 48 representative clusters according to their respective trends to reduce the dimensionality of the data set. Single representative proteins were then selected for each group. Hierarchical clustering (Figure 6B) and PCA were conducted across LNPs with various PEG-lipid types (Figure 7A, left), and LNPs formulated with similar PEG-lipids clustered together, confirming our previous findings. Relabeling PCA components with PEG percentages indicated that corona patterns remained highly dependent on the PEG-lipid type across anchors in PC1/PC2 plots (Figure 7A, right and Figure 7B). Consistent with this, Figure s3 shows that the relative abundance of representative proteins also varies with PEG‑lipid ratio. Note that the LNP surface is a mixed lipid environment (PEG-lipids, DSPC, cholesterol, and ionizable lipid), not PEG-lipid alone. Consequently, anchor- and PEG-dependent effects occur within a composite membrane whose phase behavior and ordering can influence protein adsorption. Helper lipids, in particular, differ in phase-transition temperatures (e.g., DSPC has a relatively high Tm), which might bias local surface states from fluid to gel under certain compositions and temperatures, and proteins are known to interact differently with fluid versus gel phases. Nevertheless, this hypothesis required further investigation.

**Figure 7:**
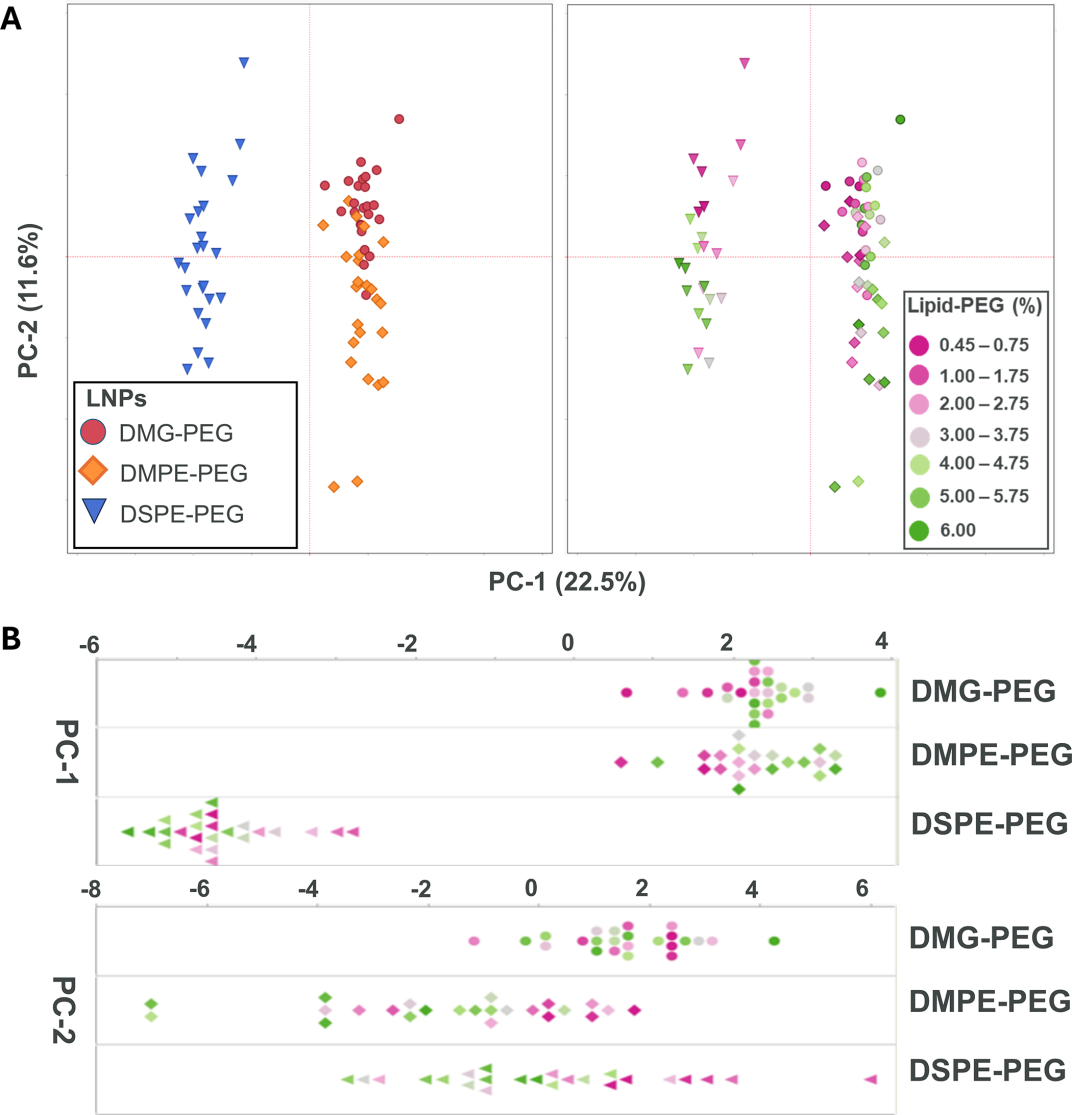
Analysis of PEG content effects on observed corona clusters. (A) PCA of representative protein corona profiles from Figure 6B, where each point represents one LNP formulation. Data points are labeled according to the PEG-lipid anchor type (left) and molar percentage (right). (B) PC loading plots with colors indicating the PEG-lipid percentages.

For the next step, we used high-throughput cellular assays to test whether PEG-dependent shifts in specific corona proteins correlate with expression efficiency. High-content imaging was used to make quantitative assessments, at the cellular level, of particle uptake, endosomal remodeling, and functional cargo delivery in terms of expressed fluorescent protein. DSPE-PEG LNPs showed a monotonic decrease in uptake with increasing PEG content, consistent with enhanced steric hindrance; by contrast, DMG- and DMPE-PEG LNPs did not exhibit linear relationships between the PEG level and uptake (Figure s4A) or endosomal remodeling (Figure s4B).

The cargo expression efficiency (GFP production per internalized particle) across four cell lines (16HBE lung epithelium, Huh7 hepatocellular, HeLa cervical epithelium, and SH-SY5Y neuroblastoma; Figure 8 panels A–D, respectively) revealed that DMG and DMPE-PEG anchors exhibit nonmonotonic response curves to increasing amounts of PEG-lipid anchors in LNPs (Figure 8, left panels). These response patterns were not attributable to cytotoxicity, as the proportion of condensed/distressed cells remained comparable across all LNPs (Figure s4C,D). SH-SY5Y showed the same qualitative trend but with markedly lower expression levels compared to other cell lines despite adequate uptake (Figure s4A). Neuroblastoma cells can internalize LNPs, but this typically results in less endosomal remodeling and increased trafficking to late endosomes, which could limit mRNA release and protein translation.^29,30^

**Figure 8:**
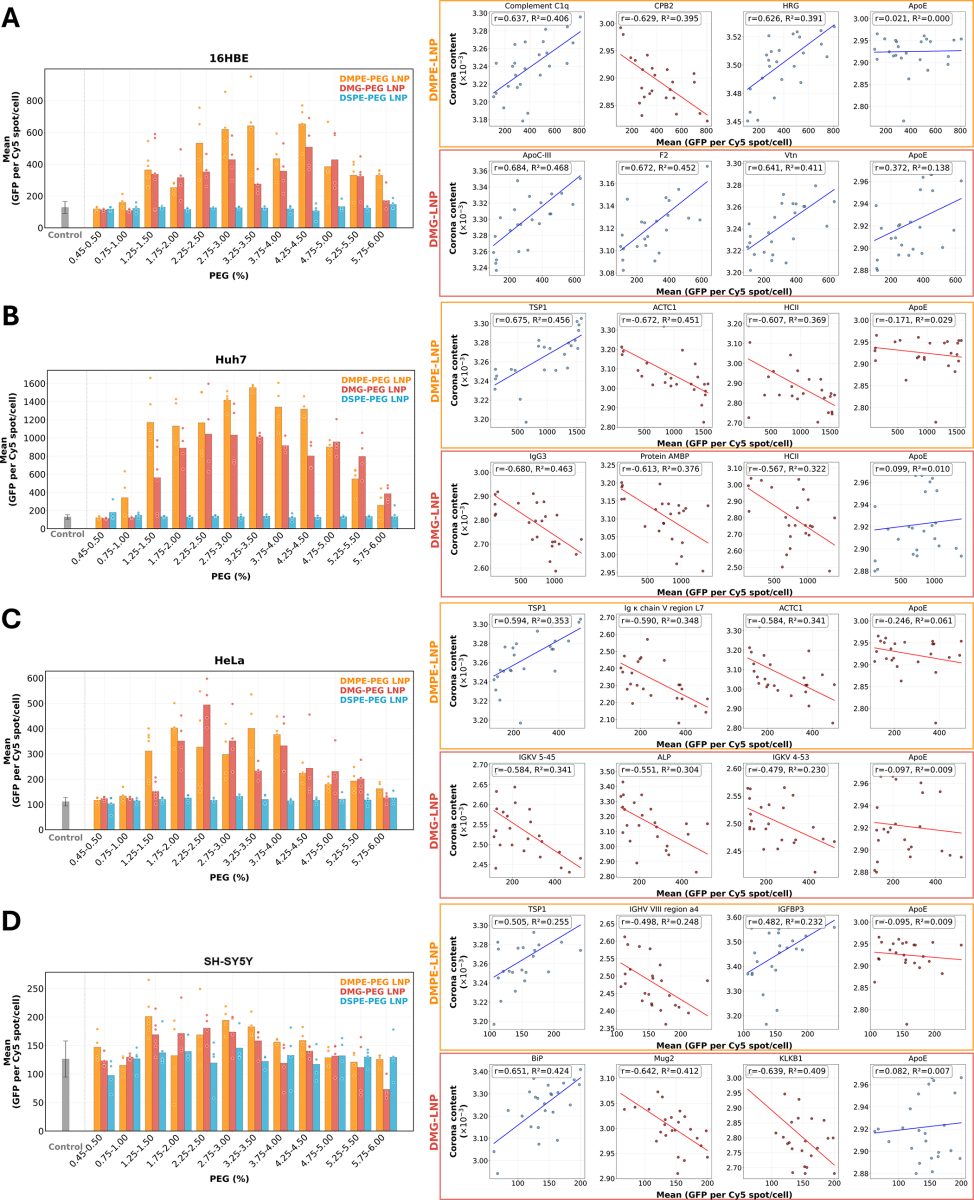
Quantitative effects of PEG-lipids and corona proteins on cell function. (A–D) Cell expression efficiency (GFP per Cy5 spot) in 16HBE, Huh7, HeLa, and SH-SY5Y. For each PEG percentage (0.25% interval), *n* = 2 per condition; PEG percentages were merged from two regimens per bar; PBS controls: *n* = 84. (A–D) Top three corona proteins per PEG-lipid anchor and cell line identified by Pearson correlation with expression efficiency in (A) 16HBE, (B) Huh7, (C) HeLa, and (D) SH-SY5Y. ApoE was highlighted in each panel. Blue lines indicate positive correlations; red lines indicate negative correlations; *r* denotes the Pearson correlation coefficient, and *R* ^2^ denotes the variance explained.

This nonmonotonic pattern in the functional data was consistent across cell lines and the sheddable DMG and DMPE PEG-lipid anchors. Below ∼1 mol %, both LNP uptake and cargo expression efficiency were low, implying poor cellular uptake of these oversized and unstable LNPs. For PEG-lipid levels above ∼5 mol %, uptake was preserved but expression per internalized particle declined, plausibly because dense PEG brushes impede post entry steps by slowing PEG shedding, stabilizing the nanoparticles, and sterically masking protonatable/fusogenic groups, with relatively less endosomal remodeling as a result (Figure s4 reveals that while uptake improves with high PEG-lipid content, endosomal remodeling occurs at approximately the same level). Consequently, the PEG-lipid content exerted a nonmonotonic effect on functional delivery *in vitro* that is not explained by uptake alone. An intermediate window (∼3–3.5 mol % in our conditions) maximized expression efficiency for sheddable anchors (DMG-PEG and DMPE-PEG). In brief, too little PEG compromises colloidal stability and results in large particles, while too much PEG likely limits mRNA release. The optimal PEG-lipid content is therefore a trade-off between particle stability and size and cargo delivery.

To connect cellular outcomes with corona composition, we asked which individual corona proteins correlate most strongly with expression efficiency across PEG percentages (noting that these are not necessarily the most abundant proteins). Given the poor protein expression mediated by DSPE-PEG LNPs, the correlation analyses focused on the data from DMG-PEG- and DMPE-PEG-lipid LNPs. We computed Pearson correlations between abundance data for each protein and single-cell GFP-per-internalized-LNP mean values, for each lipid anchor and cell line (Figure 8, right panels). The top three most correlated proteins, stratified by anchor and cell type, were identified and quantified (Figure s6). ApoE, widely viewed as an essential corona protein for LNP uptake, is shown in each panel for comparison. We believe that ApoE is one of the key proteins for cellular uptake but functional delivery in complex biological systems is governed by multivariate corona signatures and lipid composition, rather than a single protein, consistent with recent studies.^9,20^

With LNPs formulated using different PEG anchors and ratios, we sought to probe how features of the *in vitro* corona might relate to other *in vitro* outcomes, acknowledging that these associations may be context-dependent. Thrombospondin-1 (TSP1), for example, is a multifunctional adhesive ligand that engages integrins (αvβ3/αvβ5) and CD47 (positively expressed Huh7 and HeLa cells),^31–34^ potentially providing routes for productive internalization and trafficking. Conversely, immunoglobulin species, including immunoglobulin gamma-3 (IgG3), immunoglobulin heavy-chain V–III region 4 (IGHV V–III region 4), immunoglobulin kappa variable 5-45 (IGKV 5-45), and immunoglobulin kappa variable 4-53 (IGKV 4-53), bind Fc receptors,^35,36^ potentially steering uptake toward endolysosomal degradation or clearance rather than productive protein expression.

Cell type-specific correlations can also highlight distinct receptor interactions and represent another layer of complexity. As one well-established example, VTN binds αvβ5/αvβ3 integrins with the urokinase receptor (uPAR/CD87) to facilitate expression. The urokinase receptor is highly expressed in bronchial epithelial cell lines, such as 16HBE.^37^ These mechanisms align with prior observations, including the association of VTN with lung tropism of the DMG-PEG LNP group (Figure 3A).

Taken together, the PEG-lipid anchors and LNP PEG levels can program corona compositions that potentially interact with profunctional receptor pathways. While further studies are needed to strengthen this evidence, this mechanistic view explains the bell-shaped dependence of LNP cargo expression on PEG content for sheddable anchors (DMG-PEG and DMPE-PEG) and points to anchor selection, PEG percentage, and corona engineering as determinants for driving LNPs toward productive pathways.

## Conclusions

PEG-lipid anchors are active determinants of lipid nanoparticle characteristics, creating distinct surface properties, corona compositions, and pharmacological responses despite their low molar fraction. Comparisons of SPARTA^Ⓡ^, proteomic profiling, high-throughput cell assays, and *in vivo* readouts revealed clear structure–activity relationships: DMPE-PEG consistently enhanced early hepatic expression, DMG-PEG produced similar physicochemical signatures but tended to recruit more coagulation factors and vitronectin, coinciding with modest shifts in cellular functionality, and DSPE-PEG dampened early function consistent with prolonged PEG retention and cholesterol-enriched surfaces. *In vitro* expression efficiency for sheddable anchors (DMG/DMPE) followed a bell-shaped dependence on PEG content, and there was good correlation with specific protein corona components, defining an operational window for LNP composition that can balance extracellular stability with intracellular release. These findings provide a physical–molecular framework to rationally select PEG-lipid anchors and PEG levels to predefine corona–receptor engagement and tissue tropism, reducing the need for empirical screening and enabling more targeted and efficacious LNP designs.

## Methods

### LNP Formulation

The LNP formulation consisted of DLin-MC3-DMA, cholesterol, DSPC, and one of three PEG-lipids: DMG-PEG2000 (GM-020EX, NOF Corporation), DMPE-PEG2000 (PM-020CN, NOF Corporation), or DSPE-PEG2000 (DSPE-020CN, NOF Corporation). The molar ratios of these components were systematically varied within the ranges specified in Figure s5, while maintaining a constant nitrogen-to-phosphorus (N:P) ratio of 6:1 across all formulations. mRNA payloads were dissolved in 50 mM citrate buffer (pH 3.0, TekNova) to constitute the aqueous phase. The lipid components were dissolved in ethanol to form the organic phase. The two phases were combined by using a proprietary high-throughput LNP formulation device (WO2024211518A1). The flow ratio between the lipid phase (ethanol) and mRNA phase (aqueous buffer) was maintained at 1:3. For *in vitro* functional assays, LNPs were loaded with a combination of 90% eGFP-encoding mRNA (L7201, Trilink Biotechnologies) and 10% Cy5-labeled eGFP-encoding mRNA (L-7701, Trilink Biotechnologies). For *in vivo* biodistribution studies, LNPs were formulated with composition ratios of DLin-MC3-DMA:cholesterol:DSPC:PEG-lipid (50:38.5:10:1.5 molar ratio) encapsulating FLuc mRNA (L7602, Trilink Biotechnologies) at an N:P ratio of 6:1.

### LNP Characterization

The encapsulation efficiency (EE), mRNA concentration, and particle size were assessed as previously described.^9^ A RiboGreen RNA Assay (Thermo Fisher Scientific) and 1% Triton X (Sigma-Aldrich) were used to determine the total amount of mRNA present and the EE% as follows:

$$\mathrm{EE}\;(\%)=1-\left(\frac{\mathrm{mRNA}\;\mathrm{quantity}\;\mathrm{without}\;1\%\;\mathrm{Triton}\;X}{\mathrm{mRNA}\;\mathrm{quantity}\;\mathrm{with}\;1\%\;\mathrm{Triton}\;X}\right)\times 100$$

The particle size distribution and PDI of LNPs were determined using DLS on a DynaPro Plate Reader III system (Wyatt Technology) equipped with DYNAMICS 8 software (Wyatt Technology). Measurements were performed at 25 °C using standardized acquisition parameters: 4 s acquisition time per measurement with 10 consecutive acquisitions per sample. The *z*-average diameter was calculated from the autocorrelation function using the cumulants method. Each sample was analyzed in triplicate.

### Cell Imaging Experiments and Quantification

Reporter cell lines expressing mCherry-Galectin9 fusion protein were established and maintained according to previously described protocols.^38^ For *in vitro* functional assessment of LNPs, cells were seeded in 384-well CellCarrier Ultra plates (PerkinElmer, 6007558) and cultured in medium for a minimum of 16 h to achieve optimal adherence and density. Prior to LNP treatment, cells were washed three times with PBS to remove residual serum components. LNP formulations were prepared in 384-well source plates (Greiner Bio-One, 781280) and preincubated with media containing mouse serum for 4 h. The LNP solutions were then transferred to cell-containing plates. Following a 24 h LNP treatment period, cells were washed with PBS to remove unbound nanoparticles and subsequently fixed with 4% paraformaldehyde solution. Nuclei were counterstained with Hoechst 33342 (0.5 μg/mL in PBS). Multiparametric high-content imaging was performed using a CV7000 automated confocal microscope system (Yokogawa Electric Corporation) equipped with a 20× objective (numerical aperture 0.75). The fluorescence acquisition employed laser (emission filter) combinations: 405 nm (BP445/45 nm), 488 nm (BP522/35 nm), 561 nm (BP600/37 nm), and 640 nm (BP676/29 nm). Image processing and feature extraction were performed using Signals Image Artist 1.3 software (Revvity).

### LNP Corona Isolation

Protein corona formation on LNPs was investigated using an immunomagnetic separation approach established by our group.^9^ Briefly, LNP formulations were separately incubated in cell culture media supplemented with 1% mouse serum (Merck, M5905) at a concentration of 4 μg/mL mRNA (corresponding to a 200 ng total mRNA dose) for 4 h. Proteomic results obtained from duplicate LNP aliquots separately exposed to serum were later averaged. The corona isolation protocol was executed using a KingFisher Flex automated magnetic processor (Thermo Fisher Scientific) equipped with a 96-position magnetic head array. The corona complexes were isolated using M-270 Epoxy Dynabeads (Thermo Fisher Scientific, #14321D) functionalized with monoclonal anti-PEG antibodies (Merck, MABS1963) according to the manufacturer's specifications. The captured bead-LNP-corona complexes underwent sequential washing steps with PBS to remove nonspecifically bound proteins, followed by elution using a solution containing 0.5 M NH_4_OH and 0.5 mM EDTA.

### Proteomic Data Processing and Analysis

For proteomic characterization, protein denaturation, reduction, and alkylation were performed simultaneously by incubating samples with a mixture containing 10 mM TCEP (Thermo Fisher Scientific, #77720) and 20 mM 2-chloroacetamide (Merck, #22790) in 50 mM Tris buffer at 90 °C with agitation (850 rpm) for 10 min. The processed samples were subsequently subjected to overnight enzymatic digestion with trypsin (Merck, no. EMS0006) at 37 °C. Digestion was terminated by acidification with formic acid to a final concentration of 1.1%. Peptide analysis was conducted on a Q-Exactive HF mass spectrometer (Thermo Fisher Scientific) interfaced with an Evosep One liquid chromatography system (Evosep Biosystems). Raw mass spectrometry data were processed by using MaxQuant software (version 2.4.11.0). Protein identification was conducted using the Uniprot FASTA database (mouse UP000000589). Search parameters included variable modifications (N-terminal acetylation and methionine oxidation) and fixed modifications (cysteine carbamidomethylation). The false discovery rate was set to 1%, with a minimum peptide length of seven amino acids. Tryptic specificity was defined as cleavage after lysine and arginine residues with an allowance for up to two missed cleavages. Mass tolerance was set to 6 ppm for the precursor ions and 20 ppm for the fragment ions. The “match between runs” algorithm was enabled to maximize identification consistency across the sample set. Protein quantification employed both the LFQ and iBAQ algorithms. Perseus (version 2.1.5), Qlucore Omics Explorer (version 3.9), and JMP 18 (SAS Institute, Inc.) were used for data analysis (see statistical methods for more information).

### Single-Particle Automated Raman Trapping Analysis

Molecular fingerprinting of LNPs was performed using single-particle automated Raman trapping analysis (SPARTA^Ⓡ^ Biodiscovery) technology with a 785 nm laser. For each analysis, LNPs were diluted in PBS. The SPARTA^Ⓡ^ system was configured to attempt 200 individual particle acquisitions per replicate (*n* = 2), with each successful trap yielding a Raman spectrum collected over a 10 s acquisition time. Spectral processing was performed using the SPARTA^Ⓡ^ Discovery software package (version 1.1.0). Initially, the buffer contribution was minimized by subtracting 95% of the averaged PBS spectrum from each particle spectrum. The resulting data were truncated to focus on the fingerprint region (600–1800 cm^–1^) containing the most distinctive biomolecular information. Baseline correction was implemented using a Whittaker filter (smoothness log_10_: 7; differential order: 2) to remove broad fluorescence background while preserving spectral features. Signal-to-noise enhancement was achieved through the application of a second-order Savitzky–Golay smoothing algorithm. Finally, all spectra were normalized by the area under the curve to directly compare between particles of different sizes.

### Small-Angle X-ray Scattering

Structural analysis of selected LNPs samples was performed using small-angle X-ray scattering on a 3 GeV ring at the MAX IV synchrotron facility (Lund, Sweden). SAXS data were collected at the CoSAXS beamline equipped with a BioCUBE (Xenocs) sample loading robot and a temperature-controlled flow-through cell. The cell consisted of a quartz capillary of 1.5 mm inner diameter from Hilgenberg GmbH. The scattered intensity was recorded at 25 °C with X-ray wavelength *l* = 1 Å using an Eiger2 4M detector (Dectris). The sample-to-detector distance of 3.5 m was calibrated using silver behenate yielding the scattering vector *q* range from 0.003 to 0.3 Å^–1^. The exposure time was set to 10–20 ms, and the SAXS profile was obtained after averaging over 200–400 frames per sample. The data were normalized to the transmittance and scaled to absolute intensity using the scattering from water. The scattering profiles presented were background-subtracted, where the background corresponds to the LNP buffer measured directly before each sample. The particles were concentrated for these measurements to 5–6 mg/mL of total lipids using Amicon ultracentrifugation filters. The data analysis was performed using MATLAB software (R2021b).

### Molecular Dynamics Simulation

The Martini 3 force field was used for all simulations, and the three PEGylated lipids were constructed using previously developed fragments.^39,40^ Phospholipid parameters were as previously reported.^41^ The PEG-to-lipid bilayer interaction strength was reduced, and the interaction between the Q5 and SN3r beads was adjusted to 3.0 kJ/mol to reproduce atomistic bilayer affinities.

The bilayer systems were constructed using Insane^42^ and Polyply.^40^ All systems were simulated in GROMACS 2021 and minimized and relaxed using the same protocol: a steepest descent minimization of 500 steps followed by a relaxation of 20 ns using a 10 fs time step and a constant temperature (310 K) and pressure (1 bar), using a Berendsen thermostat and barostat, respectively, with semi-isotropic pressure coupling with tau of 1 (temperature) and 12.0 ps (pressure). Three independent runs reaching 1 μs, with a time step of 20 fs, were performed. The thermostat and barostat used were v-rescale and c-rescale, respectively, with tau set to 1 and 12.0 ps, respectively. During both relaxation and production runs, the compressibility was set to 3 × 10^–4^ bar^–1^. The reaction field method was used to treat electrostatics, while van der Waals interactions were truncated after 1.1 nm. The Verlet neighbor cutoff settings were adapted to avoid artifacts.^43^ Results were visualized using matplotlib in Python. The 2D number densities were calculated by using a Python script, which applies numpy. The radial distribution functions were calculated using GROMACS tool rdf.

### *In Vivo* Experiments

Animal studies were conducted using male albino C57BL/6 mice (8–10 weeks old). All animal procedures complied with local institutional guidelines and were approved by both the AstraZeneca Ethics Committee for Animal Experimentation (PARTNER case number 4622) and the Pharmaron Institutional Animal Care and Use Committee (study number PH-AZP-IVP-2024–34, Pharmaron Beijing). Three LNP formulations were evaluated, each containing one of the following PEG-lipid variants: DMG-PEG2000, DMPE-PEG2000, or DSPE-PEG2000. All formulations were loaded with FLuc mRNA to enable the quantitative assessment of tissue-specific mRNA delivery and expression. Mice were randomly assigned to experimental groups (*n* = 3 mice per formulation) and received a single intravenous (i.v.) injection via the lateral tail vein at 0.3 mg/kg (based on the encapsulated mRNA content) in a dosing volume of 5 mL/kg. Control animals received an equivalent volume of PBS. Luciferin (5 mL/kg RediJect D-Luciferin, PerkinElmer) was administrated subcutaneously 6 h postadministration. Animals were then sacrificed by CO_2_. Target tissues, including the liver, bone marrow, lungs, and spleen, were immediately harvested, weighed, and processed for luciferase activity measurement with an IVIS Lumina III (PerkinElmer). For quantitative analysis, background luminescence was subtracted using control group values, and signals were normalized to tissue weight to account for differences in the organ mass. This normalization approach enabled a direct comparison of formulation-dependent biodistribution patterns across different tissues.

### Statistics

Statistical analysis of the processed Raman spectral data sets was conducted using R Studio (version 2023.12.1). Unsupervised multivariate analysis, including principal component analysis (PCA), was employed to identify spectral patterns distinguishing the different nanoparticle populations based on their molecular composition. Graphical representation of averaged spectra and statistical outputs was generated using GraphPad Prism software (version 9.4.0).

Comparative analyses of LNP proteomic samples were performed using Perseus (version 2.1.5), Qlucore Omics Explorer (version 3.9), and JMP 18 (SAS Institute, Inc.), with LFQ intensity values serving as the primary quantitative metric. Protein corona profiles were analyzed using one-way ANOVA with *n* = 2 biological replicates per formulation. Statistical significance was determined using appropriate multivariate analyses with a correction for multiple comparisons (Qlucore). For cell imaging data, the resulting multidimensional data set was analyzed using TIBCO Spotfire software (version 11.4) to identify correlations between formulation parameters and functional outcomes. Final data visualization and statistical analyses were conducted using JMP 18 (SAS Institute, Inc.) and GraphPad Prism (ver. 9, GraphPad Software). Additional statistical analyses were performed using GraphPad Prism software (version 9.4.0) for functional assays and biodistribution studies and Qlucore Omics Explorer (version 3.9) for proteomics data analysis. For *in vivo* functional biodistribution studies, one-way analysis of variance (ANOVA) followed by Tukey’s multiple-comparison test was employed to compare LNP formulations, with *n* = 3 animals per group. Statistical significance was defined as *p* < 0.05 for all analyses.

## Supplementary Material





## Notes

(Table s1) Physicochemical characterization of DMG-PEG, DMPE-PEG, and DSPE-PEG LNPs; (Table s2) nanoparticle sizing obtained from SAXS; (Table s3) Raman spectral assignments for lipid components and intact LNPs from SPARTA^Ⓡ^ measurements; (Figure s1) molecular dynamics simulations of PEG-lipid anchor effects on PEG distribution on LNP surfaces; (Figure s2) small-angle X-ray scattering data for LNP samples; (Figure s3) heatmaps showing correlations between representative corona proteins and PEG-lipid percentage; (Figure s4) high-content imaging analysis of cellular responses to LNPs across PEG-lipid percentages; (Figure s5) molar ratio of PEG-lipids across LNPs; (Figure s6) relative amounts of corona proteins correlating with cell function across DMG-PEG and DMPE-PEG LNPs (PDF)
DMG, DMPE, DSPE duplicated protein results (XLSX)
