# Supplementary material for: The Impact of Polyethylene Glycol Lipid Anchors on the Physicochemical Properties, Protein Corona, Function, and Biodistribution of Lipid Nanoparticles
Source: ACS Nano. 2026 Mar 18;20(12):9875–90. doi: 10.1021/acsnano.5c19757 (PMC13288917; doi:10.1021/acsnano.5c19757)
Supplement: Supplementary file 1 [file nn5c19757_si_001.pdf]

Supporting Information 1

The Impact of Polyethylene Glycol Lipid Anchors on the Physicochemical Properties, Protein Corona, Function and Biodistribution of Lipid Nanoparticles

Chuan-En Lu[1], Kai Liu[1], Audrey Gallud[1], Viktoriia Meklesh[1], Lisbeth Thorup Ravnkilde[2], Juna Santos[1], Filipa Dias Louro[1], Tasso Miliotis[3], Marco A. Alfonzo-Mendez[1], Joanna Rejman[1], Luca Panariello[4], Hanna M.G. Barriga[4, 5], Molly M. Stevens[4,6], Fredrik Höök[7], Marianna Yanez Arteta[1], Johan Ulander[2], Suzy Jones[2], Alan Sabirsh[1*]

[1] Advanced Drug Delivery, Pharmaceutical Sciences, R&D, AstraZeneca, Gothenburg 43183, Sweden

[2] Data Science & Modelling, Pharmaceutical Sciences, R&D, AstraZeneca, Gothenburg 43183, Sweden

[3] Research and Early Development, Cardiovascular, Renal and Metabolism, BioPharmaceuticals R&D, AstraZeneca, Gothenburg, Sweden

[4] Department of Medical Biochemistry and Biophysics, Karolinska Institutet, Stockholm SE-171 77, Sweden

[5] Division of NanoBiotechnology, Department of Protein Science, SciLifeLab, KTH Royal Institute of Technology, Stockholm, 17165, Sweden

[6] Department of Physiology, Anatomy and Genetics, Department of Engineering Science, Kavli Institute for Nanoscience Discovery, University of Oxford, Oxford, OX1 3QU, United Kingdom

[7] Department of Physics, Division of Nano and Biophysics, Chalmers University of Technology, Göteborg 41296, Sweden

*Correspondence: Alan Sabirsh, Advanced Drug Delivery, Pharmaceutical Sciences, R&D, AstraZeneca, Gothenburg, Sweden, e-mail: alan.sabirsh@astrazeneca.com

	PEG-lipid	MC3	Cholesterol	DSPC	N:P	Buffer	EE%	Particle size (nm)	PDI
LNP1	DMG-PEG 1.5%	50%	38.5%	10%	6	pH 3, citrate	93.8	127.7	0.25
LNP2	DMPE-PEG 1.5%	50%	38.5%	10%	6	pH 3, citrate	93.5	103.1	0.19
LNP3	DSPE-PEG 1.5%	50%	38.5%	10%	6	pH 3, citrate	92.9	105.9	0.21

Table s1. Physicochemical characterization of DMG-PEG, DMPE-PEG and DSPE-PEG LNPs. Summary of lipid composition, formulation conditions, encapsulation efficiency, hydrodynamic size in diameter and PDI for LNPs carrying Fluc mRNA.

LNPs	Radius of gyration R_g, Å	I (0), cm-1	Particle size (2*R), nm
DMG-PEG	359	15.9	92.0
DMPE-PEG	309	9.8	79.8
DSPE-PEG	312	18.6	80.6

Table s2. Nanoparticle sizing obtained from SAXS. Results of the Guinier fit to the data at low q from Guinier approximation $I(q) \sim I(0)*\exp(-q^2 R_g^2/3)$, where R_g is the root mean square distance of the scattering particle's components from its center of mass and reflects the actual distribution of mass inside the particle. From R_g the size of LNP is calculated assuming a sphere of uniform density using the relationship: $R_g = \sqrt{3/5}\,R$, where R is the physical radius of a particle.

Raman shifts (cm^{-1})	Representative Raman band (cm^{-1})	Chemical assignments	Ref.
544	501 – 576	P-O stretching	ref[1]
698	576 – 718	Cholesterol	ref[2]
766	718 – 827	C-N-C bending, ethanolamine	ref[3]
877	871 – 941	C-O skel.	ref[4]
1084	1037 – 1255	C-C twisting	ref[5]
1300	1255 – 1376	CH_2 twisting	ref[6]
1442	1376 – 1442	CH_2-CH_3 bending	ref[7, 8]
1656	-	C=C stretching	ref[9]

Table s3. Raman spectral assignments for lipid components and intact LNPs from SPARTA® measurements. Band positions, tentative molecular vibrational modes and component attributions derived from single-particle spectra.

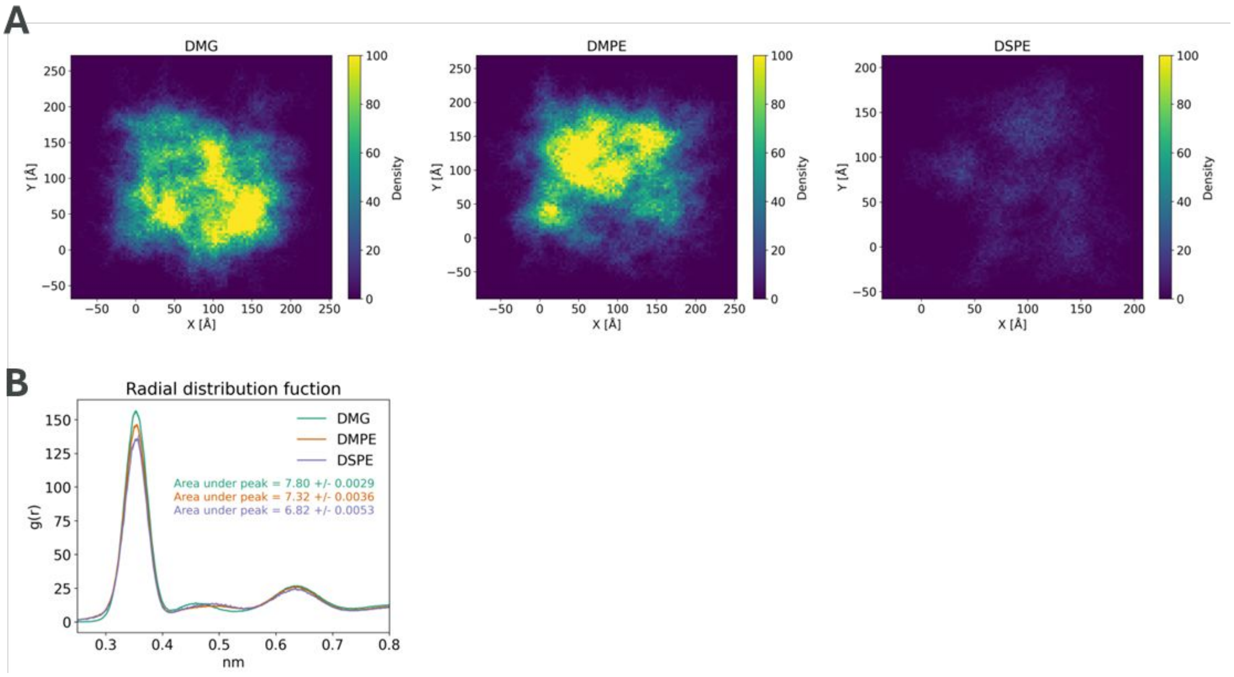

Figure s1. Molecular dynamics simulations of PEG-lipid anchor effects on PEG distribution on LNP surfaces. (A) PEG density (y-axis) of the top leaflet across the xy-plane (x-axis) within DMG-PEG, DMPE-PEG and DSPE-PEG LNPs. The plots are from one production run. (B) Radial distribution function of PEG. The area under the first peaks were shown. The error is the standard deviation across three independent runs.

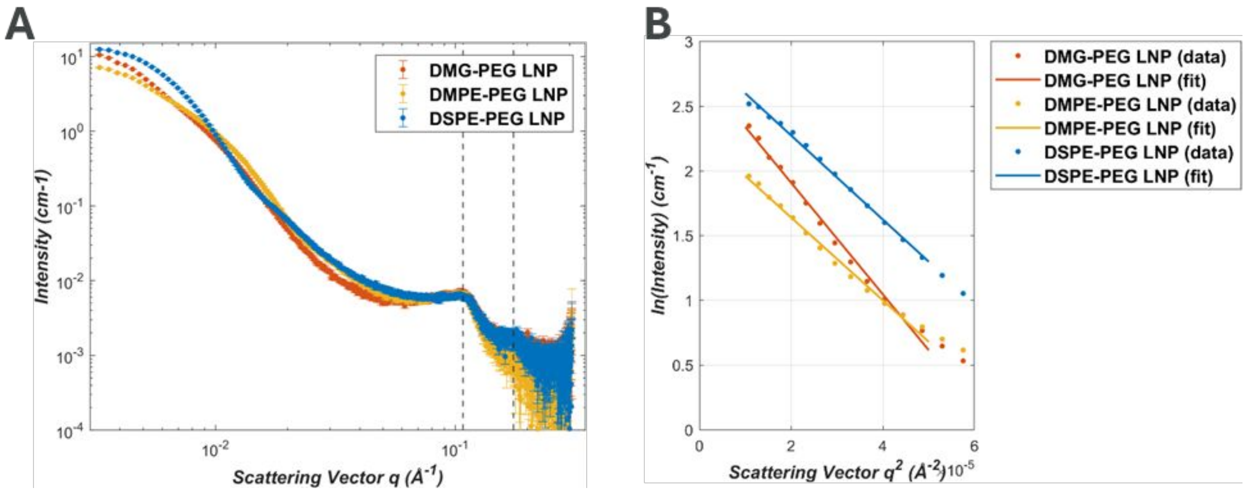

Figure s2: Small-angle X-ray scattering data for LNPs samples. (A) Comparison of the scattered intensity as a function of scattering vector reveals distinctive internal structure properties. The dashed vertical line at q = 0.108 $Å^{-1}$ corresponds to the ordered structure inside the LNP core with a repeat distance of d = 2p/q = 58 Å (in agreement with reported value in our previous literature[10]). DSPE-PEG LNP showed a shoulder at higher q values ~ 0.175 $Å^{-1}$ indicating a presence of additional structures. (B) Curve fitting on data from the lower region of the scattering profile using to obtain the size of the nanoparticles (see table s2).

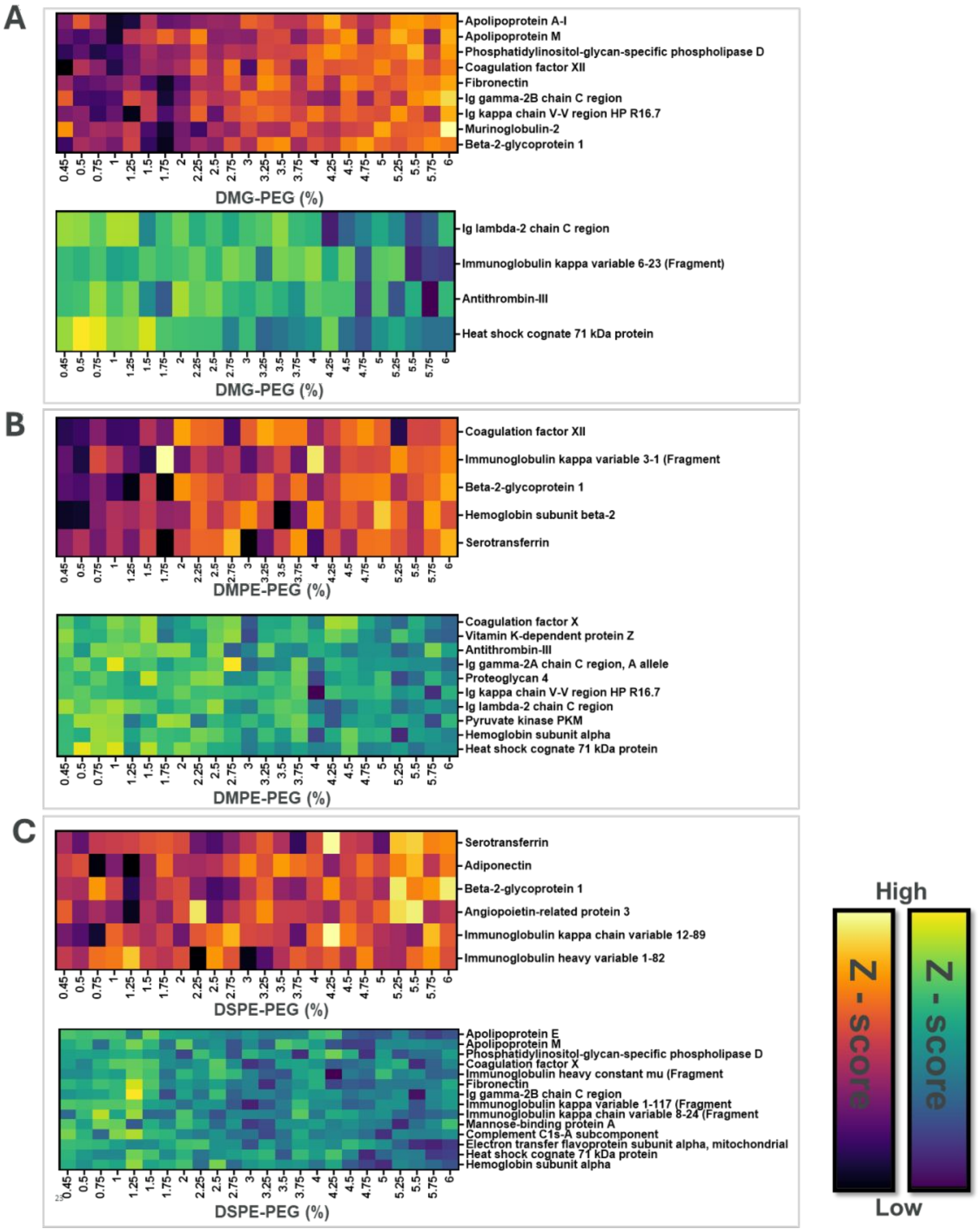

Figure s3. Heatmaps showing correlations between representative corona proteins and PEG–lipid percentage. Relative protein abundances are displayed as Z-scores: positive correlations use an orange-to-black gradient and negative correlations use a yellow-to-black gradient. Proteins were selected by Pearson correlation coefficient (r ≥ 0.4). Panels: (A) DMG–PEG, (B) DMPE–PEG, and (C) DSPE–PEG.

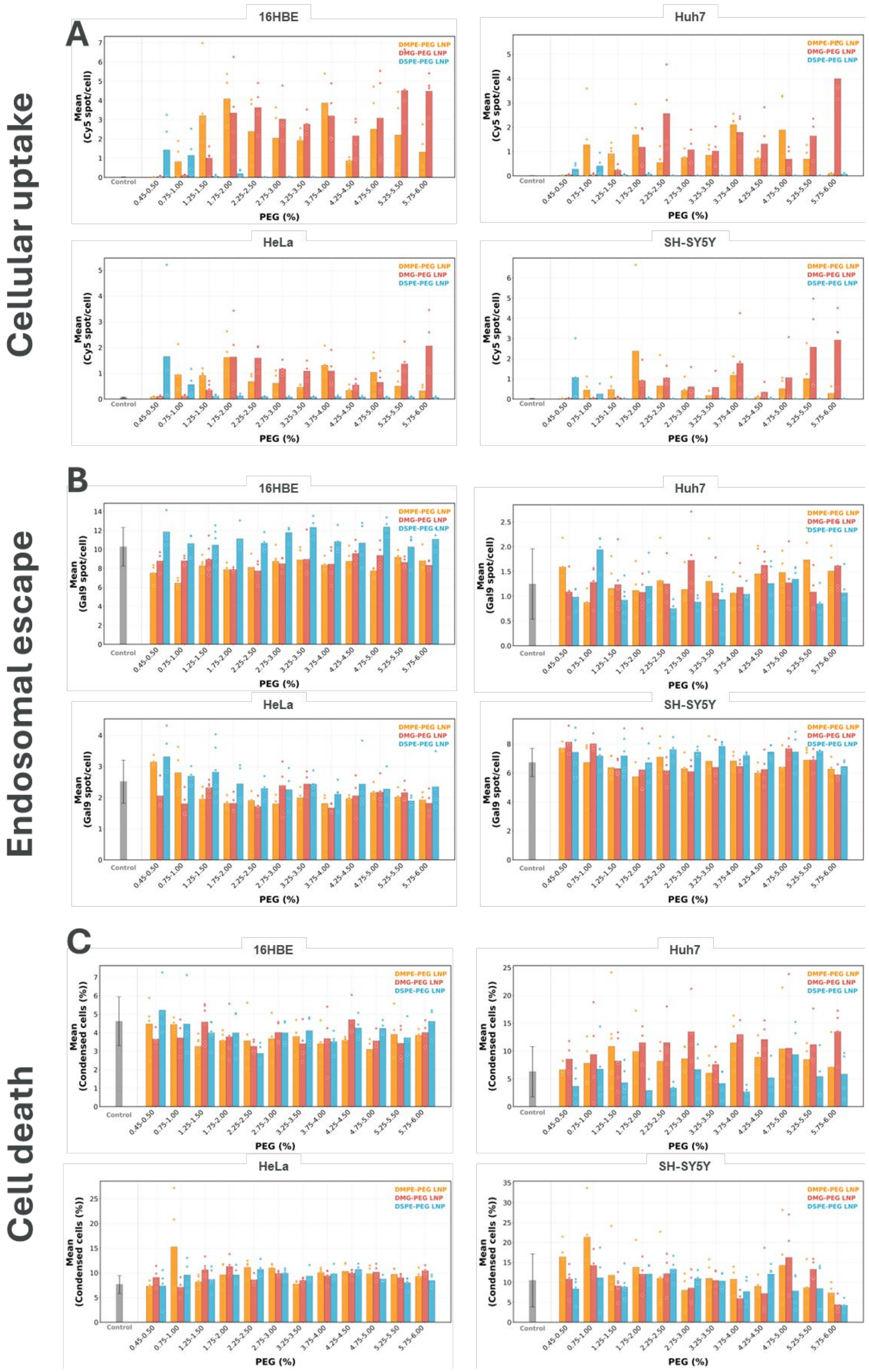
A
B
C
Cellular uptake
Endosomal escape
Cell death
16HBE
Huh7
HeLa
SH-SY5Y
DMPE-PEG LNP
DMG-PEG LNP
DSPE-PEG LNP
Mean (Cy5 spot/cell)
Mean (Gal9 spot/cell)
Mean (Condensed cells (%))
PEG (%)
Control
0.45-0.50
0.75-1.00
1.25-1.50
1.75-2.00
2.25-2.50
2.75-3.00
3.25-3.50
3.75-4.00
4.25-4.50
4.75-5.00
5.25-5.50
5.75-6.00

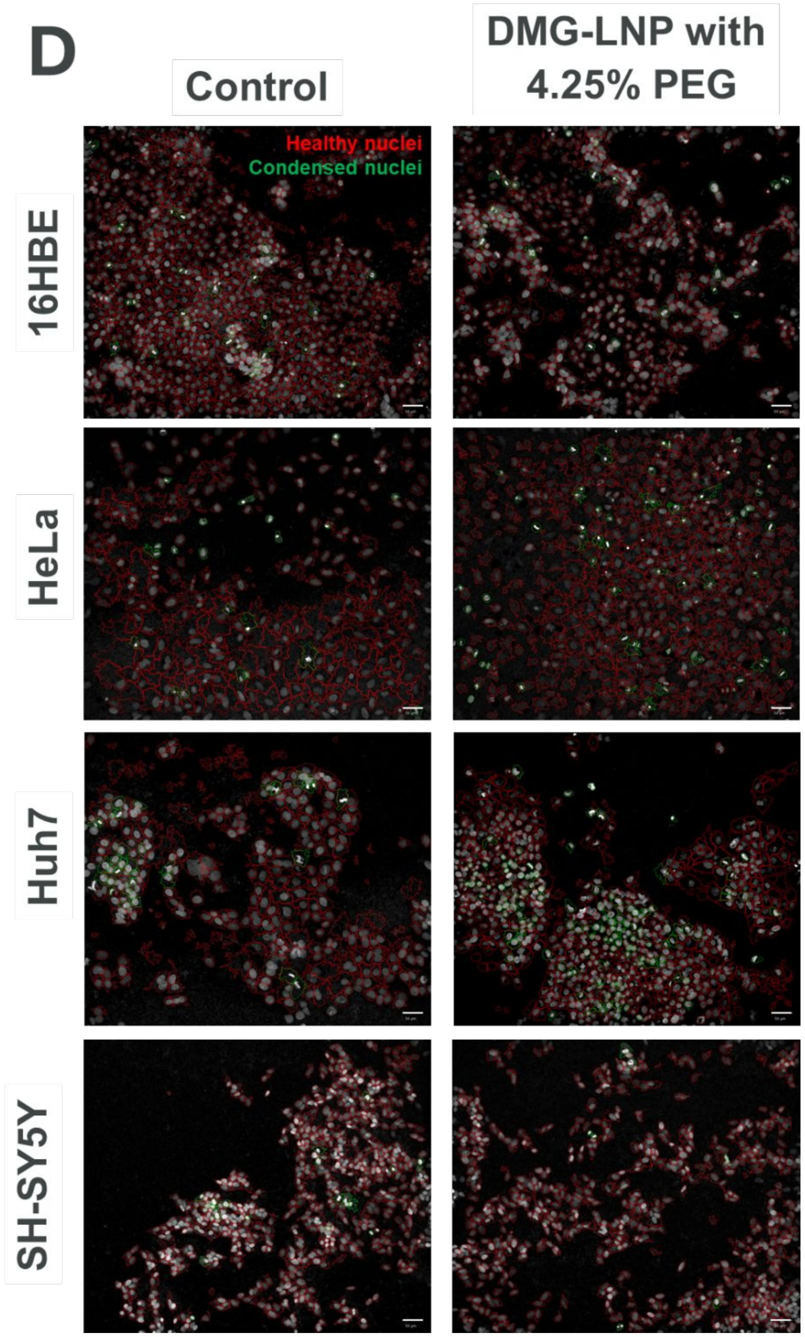

Figure s4. High-content imaging analysis of cellular responses to LNPs across PEG–lipid percentages. (A) LNP uptake (Cy5 spots per cell) across PEG levels. (B) Endosomal remodeling (galectin-9 recruitment) across PEG levels. (C) Percentage of condensed cells indicating cellular distress across all cell lines and PEG levels. For each PEG percentage (0.25% interval), n = 2 per condition; PEG percentages were merged from two regimens per bar; PBS controls: n = 84. (D) Representative confocal images acquired 24 h post-dosing for 16HBE, Huh7, HeLa, and SH-SY5Y cells treated with PBS (left) and LNPs formulated with DMG-PEG (4.25%) (right). Nuclei are shown in white color; cells with green border indicate condensed cells, filter setting: Nucleus area (μm^2) < 350 and nucleus intensity > 3000 arbitrary units; while cells with red border indicate healthy cells. Scale bars: 50 μm.

			Mean Particle size (nm)			Mean (PDI)		
	lipid-PEG(%)	DSPC(%)	DMG	DMPE	DSPE	DMG	DMPE	DSPE
1	0.45	11.05	2570	262	863	0.62	0.25	0.76
2	0.50	11.00	1256	225	264	0.85	0.26	0.21
3	0.75	10.75	595	184	213	0.44	0.13	0.17
4	1.00	10.50	667	172	175	0.65	0.14	0.12
5	1.25	10.25	523	170	167	0.37	0.23	0.16
6	1.50	10.00	183	144	193	0.17	0.15	0.17
7	1.75	9.75	163	124	176	0.21	0.17	0.19
8	2.00	9.50	152	114	169	0.19	0.15	0.12
9	2.25	9.25	136	116	157	0.17	0.17	0.15
10	2.50	9.00	121	107	148	0.20	0.20	0.16
11	2.75	8.75	140	108	163	0.22	0.21	0.19
12	3.00	8.50	134	101	154	0.21	0.22	0.15
13	3.25	8.25	110	95	167	0.20	0.20	0.27
14	3.50	8.00	131	91	139	0.20	0.20	0.15
15	3.75	7.75	117	97	135	0.21	0.20	0.20
16	4.00	7.50	130	98	132	0.26	0.17	0.16
17	4.25	7.25	117	89	127	0.22	0.19	0.15
18	4.50	7.00	114	84	105	0.21	0.21	0.27
19	4.75	6.75	101	85	115	0.23	0.18	0.16
20	5.00	6.50	112	85	112	0.21	0.18	0.25
21	5.25	6.25	124	85	124	0.20	0.20	0.19
22	5.50	6.00	129	83	103	0.21	0.20	0.17
23	5.75	5.75	101	77	106	0.21	0.19	0.31
24	6.00	5.50	94	85	94	0.22	0.19	0.22

Figure s5. Molar ratio of PEG-lipids across LNPs. Molar ratios of PEG–lipids across LNP formulations. PEG–lipid content ranges from 0.45% to 6% for DMG–PEG, DMPE–PEG, and DSPE–PEG formulations. Corresponding particle size and PDI were determined for each formulation.

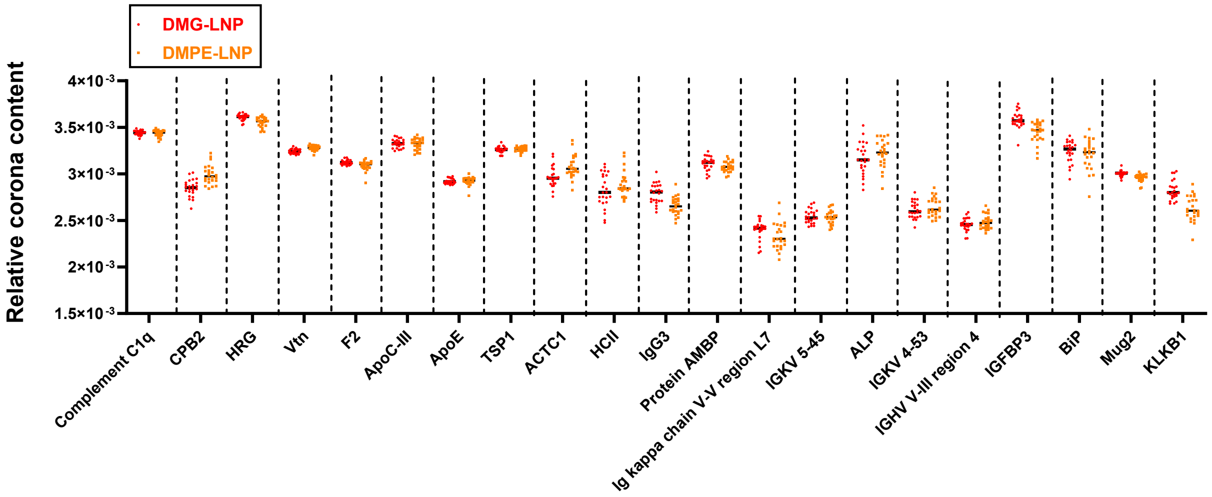

Figure s6. Relative amounts of corona proteins that correlated with cell function across DMG-PEG and DMPE-PEG LNPs from Fig. 6. Proteins were selected based on strong correlations with expression efficiency (the amount of GFP produced / the amount of mRNA delivered) in Fig. 6. Each dot represents a normalized LFQ intensity from one formulation within the indicated anchor group, DMG-PEG or DMPE-PEG and a series of PEG percentages. Black horizontal bars represent the group mean for each anchor type.

References

(1) Vivekanandan, K.; Selvasekarapandian, S.; Kolandaivel, P.; Sebastian, M. T.; Suma, S. Raman and FT-IR spectroscopic characterisation of flux grown KTiOPO4 and KRbTiOPO4 non-linear optical crystals. *Materials Chemistry and Physics* 1997, *49* (3), 204-210.
(2) Czamara, K.; Majzner, K.; Pacia, M. Z.; Kochan, K.; Kaczor, A.; Baranska, M. Raman spectroscopy of lipids: a review. *Journal of Raman Spectroscopy* 2015, *46* (1), 4-20.
(3) Suo, L.; Zheng, F.; Hu, Y.-S.; Chen, L. FT-Raman spectroscopy study of solvent-in-salt electrolytes. *Chinese Physics B* 2015, *25*, 016101.
(4) Stengelin, E.; Kuzmina, A.; Beltramo, G. L.; Koziol, M. F.; Besch, L.; Schröder, R.; Unger, R. E.; Tremel, W.; Seiffert, S. Bone Scaffolds Based on Degradable Vaterite/PEG-Composite Microgels. *Advanced Healthcare Materials* 2020, *9* (11), e1901820.
(5) Pant, U.; Tate, J.; Liu, X.; Birse, N.; Elliott, C.; Cao, C. From automated Raman to cost-effective nanoparticle-on-film (NPoF) SERS spectroscopy: A combined approach for assessing micro- and nanoplastics released into the oral cavity from chewing gum. *Journal of Hazardous Materials* 2025, *486*, 136978.
(6) Czernuszewicz, R. S.; Rankin, J. G.; Lash, T. D. Fingerprinting Petroporphyrin Structures with Vibrational Spectroscopy. 4. Resonance Raman Spectra of Nickel(II) Cycloalkanoporphyrins: Structural Effects Due To Exocyclic Ring Size. *Inorganic Chemistry* 1996, *35* (1), 199-209.
(7) Lin-Vien, D.; Colthup, N. B.; Fateley, W. G.; Grasselli, J. G. CHAPTER 2 - Alkanes. In *The Handbook of Infrared and Raman Characteristic Frequencies of Organic Molecules*, Lin-Vien, D., Colthup, N. B., Fateley, W. G., Grasselli, J. G. Eds.; Academic Press, 1991; pp 9-28.
(8) Lu, C.-e.; Levey, R. E.; Ghersi, G.; Schueller, N.; Liebscher, S.; Layland, S. L.; Schenke-Layland, K.; Duffy, G. P.; Marzi, J. Monitoring the macrophage response towards biomaterial implants using label-free imaging. *Materials Today Bio* 2023, *21*, 100696.
(9) Samek, O.; Jonas, A.; Pilat, Z.; Zemanek, P.; Nedbal, L.; Triska, J.; Kotas, P.; Trtílek, M. Raman Spectroscopy for the characterization of algal cells. *Proc SPIE* 2010, *7746*.
(10) Yanez Arteta, M.; Kjellman, T.; Bartesaghi, S.; Wallin, S.; Wu, X.; Kvist, A.; Dabkowska, A.; Székely, N. K.; Radulescu, A.; Bergenholtz, J.; Lindfors, L. Successful reprogramming of cellular protein production through mRNA delivered by functionalized lipid nanoparticles. *Proceedings of the National Academy of Sciences* 2018, *115*.
